# Rules of incidental operation risk propagation in metro networks under fully automatic operations mode

**DOI:** 10.1371/journal.pone.0261436

**Published:** 2021-12-16

**Authors:** Wenying Chen, Jinyu Yang, Mohammad T. Khasawneh, Jiaping Fu, Baoping Sun

**Affiliations:** 1 School of Management and Engineering, Capital University of Economics & Business, Beijing, China; 2 Beijing Key Laboratory of Megaregions Sustainable Development Modeling, Beijing, China; 3 Department of Systems Science and Industrial Engineering, State University of New York at Binghamton, Binghamton, New York, United States of America; Ningbo University, China, UNITED STATES

## Abstract

The frequent interruptions of network operation due to any incident suggest the necessity to study the rules of operational risk propagation in metro networks, especially under fully automatic operations mode. In this study, risk indicator computation models were developed by analyzing risk propagation processes within transfer stations and metro networks. Moreover, indicator variance rules for a transfer station and different structural networks were discussed and verified through simulation. After reviewing the simulation results, it was concluded that under the impacts of both sudden incident and peak passenger flow, the more the passengers coming from platform inlets, the longer the non-incidental line platform total train operation delay and the higher the crowding degree. However, train headway has little influence on non-incidental line platform risk development. With respect to incident risk propagation in a metro network, the propagation speed varies with network structure, wherein an annular-radial network is the fastest, a radial is moderately fast, and a grid-type network is the slowest. The conclusions are supposed to be supports for metro operation safety planning and network design.

## Introduction

### Background

Rapid urbanization in China has greatly boosted the initiation of increased metro rail transit projects throughout the nation. By 2017, 34 cities had 165 rail transit lines in service of which the total length is 5033 km [1]. However, along with service on numerous metro lines come not only the benefits of road traffic relief and environmental pollution reduction but also new challenges and demands for reliable and safe metro operation [2]. When it comes to network operation, the relations among different lines and stations are complex, wherein an incidental risk could be propagated in the entire rail transit network as a result of a local fault or a failure. If a local incident cannot be removed in a timely manner, it will lead to negative effects such as passenger retention, train delay, and cessation of metro line service, and potentially even more serious casualties. For example, on November 22, 2009 a crash occurred on Metro Line 1 in Shanghai because of equipment failure, in addition to anxiety among passengers, this failure caused all the metro lines to fail for five hours. Another example is that a mass of train delays happened on Metro Line 10 in Beijing owing to a signal fault, leading to the cessation of 101 trains and disturbing more than 200,000 people [3]. Therefore, rules of incidental operation risk propagation through metro network are needed to study.

With the rapid development of Artificial Intelligence (AI), fully automatic operations have become prevalent. Such operational mode has been already adopted for Shanghai Metro Line 10, the Beijing Subway Line Yanfang, and several other lines in China. Compared to traditional modes of operation, the safety risk arising from driver behavior in automated modes could be reduced and operational efficiency can be improved. This can be attributed to the full-automatic functions of line operation, in and out of the station, that can be accomplished. However, emergency response capacity might be impaired under this mode. When a train in the line has to be shut off as a result of an incident, such as faulty equipment, following trains will have to apply brakes to cease successively dominated by Automatic Train Protection (ATP). If the faulty train cannot be removed in time, other trains on the line will be suspended for a period. The incident risk will spread to other lines through transfer stations leading to a cessation of the entire network operation.

### Literature review

Several studies discussed incidental risk for transportation operations such as Xu et al. [4], and Ahmad and Khan [5]. Kyriakidi et al. [6] analyzed accident data from 2002 to 2009 and developed a comprehensive assessment using a safety maturity model for the metro operation. Louie et al. [7] focused on the effects of the incidents’ location and occurrence time, the train-type involved, and the non-adherence to recovery procedures in an effort to investigate the relationship between those factors and the resulting delay duration. Zhang et al. [8] introduced details of all incidents that have occurred in metro operations to prevent accidents using an adaptable incident database. Also, Lu et al. [9] introduced a framework that incorporates metro accident causes, processes, and solutions to prevent accidents using a new case-based reasoning method. In road safety and automatic driving aspect, lane-changing models are developed to simulate the off-ramp behaviors for autonomous vehicles and the findings can provide useful references for the management of the automated highway systems [10]. Zheng et al. [11] proposed a cooperative lane changing strategy to improve traffic operation and safety at a diverging area nearby a highway off-ramp in an environment with connected and automated vehicles. Guo et al. [12] developped traffic conflict-based real-time safety models for signalized intersections and the models have potential applications in real-time safety evaluation. Li et al. [13] proposed a machine learning method for the short-term prediction of lane-changing impacts during the propagation of traffic oscillations and proved the method is useful for safety maximization. Zhang et al. [14] conducted a simulation study to provide some practical suggestions for using propensity score method in road safety evaluations. Wang et al. [15] reviewed surrogate safety measures and their applications in connected and automated vehicles safety modeling studies.

In spite of the safety analysis of operations as discussed above, the current research on metro network risk propagation focuses on two aspects: 1) network reliability and destroy-resistant ability and 2) sudden massive passenger flow and congestion propagation.

Gu et al. [16] review studies on transportation network performance under perturbations. Three concepts were discussed: reliability, vulnerability, and resilience. As far as metro network reliability is concerned, there is limited research in this area. The concept of transportation network connectivity reliability is based on maintaining the connectivity probability between two points in a transportation network, which was issued originally by Mine and Kawai [17]. Iida and Wakabayashi [18] and Sanso and Soumis [19] used computation method of minimal path set and cut set for road network terminal reliability and applied the shortest path computation method for network connectivity reliability. Latora and Marchiori [20] discussed the center point of a metro network by studying the Boston metro and came up with the concepts of global efficiency and component efficiency. Crucitti et al. [21] showed classical network connectivity variance after random and deliberate attacks by evaluating network efficiency. On the other hand, A few attempts have been made to study metro network destroy-resistant ability. Angeloudi and Fisk [22] found that a metro network have higher survivability against a random attack compared to relatively lower survivability in the case of a deliberate attack. Wang et al. [23] concluded that the vulnerability is apparent when a metro station is attacked deliberately while the robustness is obvious when station-to-station tunnels are assaulted in the same manner. Yang et al. [24] revealed that the Beijing subway system exhibits characteristics of a scale-free network, with relatively high survivability and robustness when faced with random failures. Jenelius et al. [25] focused on the importance of network edge and site in vulnerability analysis and the Beijing Subway network vulnerability was compared with Shanghai’s and it was concluded that the more complex a metro network structure, the lower its vulnerability [26].

These studies examined just the static topological structure of a metro network. The dynamic characters of transportation network have been thoroughly studied in these literatures. Wu et al. [27] proposed a choice-based framework for modelling the supply/demand interaction in a dynamically priced FFCS market. Huang et al. [28] presented an optimization model including static and dynamic parts for the network design problem of the demand-responsive customized bus. Zhang et al. [29] developped spatial-temporal adversarial network to assign the generative factors of traffic flow to the feature vector in latent space and reconstructs the high-dimensional citywide traffic flow. Authors proved that the model not only improves the prediction accuracy but also characterizes structural properties of the traffic evolution process.

Because of the confined underground space, problems related to passenger flows and their developments were addressed in the literature. Silva et al. [30] studied the effects of a station closure or interruption on passenger flow behavior and station crowdedness. Gao et al. [31] studied the number of passengers stranded at a station and proposed an optimized, iterative algorithm based on the Beijing subway network. Huang et al. [32] studied the temporal-spatial erosion process through a congestion propagation model and identified various control points. Xiao and Zhang [33] developed a dynamic congestion propagation model to account for the dynamics of disaster spread. The studies above focused on aggregation and congestion propagation rules of passenger flow caused by a large-scale activity.

Nevertheless, the effects of an incident will spread through an operating metro network under various conditions and operational modes. Incidental risk will spread to other lines through transfer stations, whose carrier is “passenger flow”. Specifically, when rush hour passenger flow overlaps with incident-caused congestion, the negative consequences on the network will result in a “snowball effect,” slowing down trains and increasing station crowding, in addition to other serious secondary accidents such as a stampede. In this research, sudden incidental risk propagation mechanism are discussed considering both static network topological structure and dynamic passenger flow. This study follows a point-line-network order starting from an incident or an emergency in a single line. Incident risk and peak passenger flow propagation along transfer stations and consequently other lines, under full-automatic operation mode, are emphasized. Risk computation models for a transfer station and metro network are developed for risk indicator variance rules.

The rest of this article is organized as follows. Section 2 presents a qualitative analysis of risk propagation through a metro network and a quantitative computational model of risk indicators. Section 3 discusses how risk indicators vary as an incidental risk propagation along metro network. Finally, Section 4 summarizes and concludes this paper.

## Method

### Risk propagation along transfer station

#### Qualitative risk analysis

In this study, a train entering an island transfer station during rush hour is used as an example for the qualitative risk analysis and quantitative risk evaluation in cases of failures due to an incident in the metro network. For convenience, the incident line is called Line 1, while the other line connecting with Line 1 by the transfer station is called Line 2. The platform of transfer station linked by Line 1 is Platform 1 and that linked by Line 2 is Platform 2.

**Fig 1:**
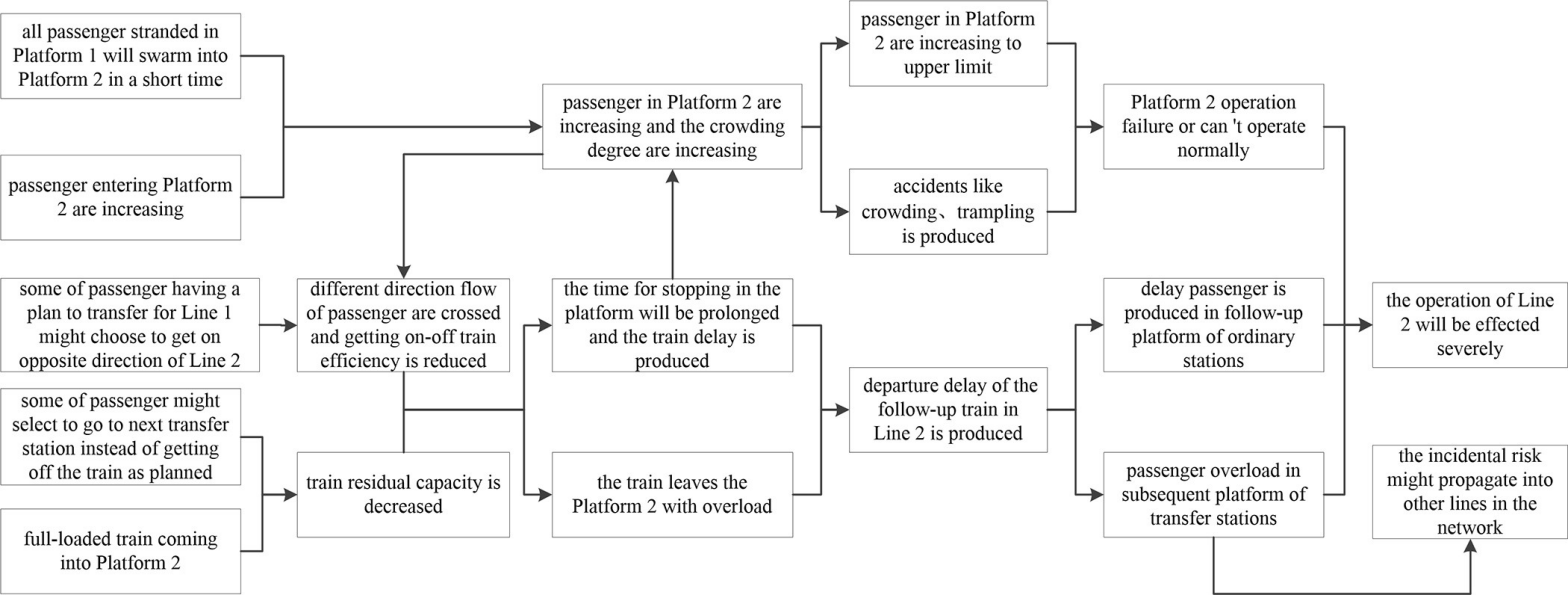
Risk propagation path of incidental operation along transfer station.

Fig 1 shows the incidental risk propagation paths along a transfer station. When Line 1 is suspended due to an incident, there are several changes of passenger flow in Platform 2: (1) all passenger stranded in Platform 1 will swarm into Platform 2 in a short period of time; (2) passenger planning to come into Platform 1 might change their minds and enter Platform 2, thereby increasing the passengers number at Platform 2; (3) some passengers planning to transfer to Line 1 might choose the opposite direction of Line 2 to pursue another transfer route (therefore, different direction flow of passenger is crossed and the number of passengers in Platform 2 increase); and (4) some passengers on Line 2 who had been planning to transfer to Line 1 might select to go to the next transfer station to pursue another travel route instead of getting off the train as originally planned.

After a fully loaded train comes to a full stop at Platform 2, its residual capacity is decreased because fewer passengers get off. In addition, unloading-loading efficiency is reduced, and stopping time at the platform increases because of the large number of passengers and the lack of order. If the stopping time at the platform is longer than the scheduled time, a train delay takes place.

As the train leaving Platform 2 is overloaded, its available capacity will be less upon arrival at the next station, thereby increasing the overall number of passengers waiting for a train at subsequent stations. Therefore, the number of delayed passengers will increase over time, leading to congestion at other stations along Line 2. Meanwhile, when the train enters subsequent transfer stations of Line 2, there will be passenger overload in these stations because they have to accept additional passengers who were supposed to have gotten off at Platform 2 of the original transfer station. Consequently, the incident risk might propagate into other lines in the network through these transfer stations connected with Line 2.

In conclusion, the primary indexes of risk propagation along transfer stations are dependent on the operational delay of Line 2 trains passing the transfer station as well as the average crowding degree of the transfer station.

#### Quantitative risk computation model

To quantify the risk propagation, several assumptions have to be made: (1) all trains in the metro network operate automatically; (2) train headway, the dwelling time fixed in the train diagram, and passenger flow-through rate, are assumed to be the same for all trains; (3) before the start of the computation process, there are no residual passengers on the train platform as trains depart the station; and (4) trains getting to and departing from a station are at maximum passenger capacity in peak hours.

*Train operation delay*. (a) ***Train departure delays of Line 2*** (*t*^*s*^_*ty*_): The operation delays of Line 2 up-trains are computed only because those of down-trains are the same. After Line 1 suspension, the Line 2 up-train arriving at Platform 2 is *b*_*j*_ (*j* = 1,2,…,*m*). The operation delay for a single train is the difference between the actual dwelling time and the dwelling time fixed in the schedule as illustrated in Eq (1). The actual dwelling time represents the duration through which the train stops at a platform to allow for the loading and unloading of passengers. If the value is less than or equal to 0, there is no delay. The total operation delay of Line 2 up-trains is the sum of each train departure delays as shown in Eq (2).


$$t_{{}_{\mathrm{fy},j}}^{s}=\max\{\frac{\min\{R_{j},K_{\max}\cdot (1-\tau )\}}{v_{c}}-t_{\mathrm{td}},0\}$$ (1)

$$t_{{}_{\mathrm{fy}}}^{s}=\sum t_{{}_{\mathrm{fy},j}}^{s}$$ (2)

Here, *t*^*s*^_*fy*_ is total operation delays of Line 2 up-train, (s); *t*^*s*^_*fy*,*j*_ is *b*_*j*_ train departure delay at transfer station, (s); *R*_*j*_ is the waiting passenger quantity when *b*_*j*_ train enters Platform 2, *K*_*max*_ is the train passenger capacity, *τ* is percentage of passenger flow, *v*_*c*_ is velocity of passenger getting on train per unit time [$\frac{people}{s}$]; and *t*_*td*_ is the dwelling time fixed in the train schedule [*s*].

(b) ***Waiting passenger quantity when train entering Platform 2*** (*R*_*j*_): When train *b*_*1*_ enters Platform 2, the initial waiting passenger quantity *R*_*1*_ is the number of arriving passengers in a train headway. On the other hand, the number of passengers waiting (*R*_*j*_*)* when *b*_*j*_ train enters Platform 2 is the sum of the residual passengers on Platform 2 when train *b*_*j-1*_ departs and the arriving passengers accumulating between *b*_*j-1*_ train leaving and *b*_*j*_ train entering. The number of residual passengers when *b*_*j-1*_ train departs is the sum of the number of passengers waiting when *b*_*j-1*_ train enters (*R*_*j-1*_) and the number of passengers transferring from the opposite direction train when *b*_*j-1*_ train is at the station, that is *K*_*max*_*(1-τ)·μ* (*μ* is the proportion of reversing passengers relative to the quantity of getting off from the opposite direction train), minus the number of passengers getting on train *b*_*j-1*_ *K*_*max*_*(1-τ)*. If the result is less than or equal to 0, the number of residual passengers when *b*_*j-1*_ train departs is 0. This can be represented in Eqs (3–4).


$$R_{1}=R_{0}=t\cdot \phi^{s}$$ (3)

$$R_{j}=\max\{0,\;R_{j-1}-K_{\max}\cdot (1‐\tau )\cdot (1‐\mu )\}+(t+t_{{}_{\mathrm{fy},j-1}}^{s})\cdot \phi^{s}$$ (4)

Here, *R*_*0*_ is the initial waiting passenger quantity [*people*]; *R*_*j*_ is the waiting passenger quantity when *b*_*j*_ train enters Platform 2 [*people*]; *t* is the formal train headway [*s*]; *φ*^*s*^ is the average velocity of arrival at the platform $\left[\frac{People}{s}\right]$.

(c) ***Velocity of passengers getting on train*** (*V*_*c*_): The velocity of passengers getting on a train is determined by the train door number, average velocity of passengers getting on one single door (*γ* $\left[\frac{People}{s}\right]$), and cross-influence coefficient of passenger flow on and off the train (*β*). Generally, in Beijing, there are six coaches in a train and four doors per coach. Therefore, there are a total of twenty-four doors per train. The velocity of passengers is represented mathematically as shown in Eq (5).


$$v_{c}=24\cdot \gamma \cdot \beta$$ (5)

(d) ***Cross-influence coefficient of passenger flow on and off train*** (*β*): The value of *β* represents the loading efficiency due to the number of passengers getting on and off the train and is primary determined by the passenger density near a train door. While passenger density is relatively low, there is no mutual interference. Hence, the duration of unloading (former) and loading (latter) are the same and *β* is 0.5. In contrast, while passenger density is too high, even exceeding the minimum limit of 0.33 [$\frac{m^{2}}{people}$] according to China Code for Design of Metro [34], that is, more than 3 $\left[\frac{people}{m^{2}}\right]$, the mutual interference is very severe. In this case, *β* is proportional to the reciprocal of the square of passenger density [35]. Therefore, the cross-influence coefficient of passenger flow on and off a train (*β*) is calculated using Eq (6).


$$\left\{ \begin{array}{l} \beta =0.5\cdots \cdots \cdots \cdots \rho_{s}\leq 3\\ \beta =0.5\cdot \frac{9}{\rho_{s}{}^{2}}\cdots \cdots \rho_{s}>3 \end{array}\right.$$ (6)

Here, *ρ*_*s*_ is the passenger density $\left[\frac{people}{m^{2}}\right]$.

*Average crowding degree at platform 2 F(T)*. (a) ***Average crowding degree at Platform 2*** (*F*(*T*)): According to Li et al. [36], *F*(*T*) is the ratio of the actual number of passengers waiting and the capacity of the platform. This is illustrated using Eq (7).


$$F(T)=\frac{\varphi \cdot T+R_{0}-P}{Z}$$ (7)

Here, *φ* is the average velocity of people arriving at the platform $\left[\frac{People}{s}\right]$; *T* is incident duration [*s*]; *R*_*0*_ is the initial number of passengers waiting [*people*]; *P* is the number of people departing when the train leaves Platform 2 [*people*]; and *Z* is the platform passenger capacity [*people*].

(b) ***The number of passengers departing when train leaves* (***P)*: *P* is the number of passengers departing when the train leaves Platform 2, including passengers getting on up-train and down-train as captured by Eq (8).


$$P=P_{}^{s}+P_{}^{x}=\sum_{j=1}^{(T-t_{\mathrm{fy}}^{s})\backslash t}\min\{R_{j}^{s},K_{\max}\cdot (1‐\tau )\}+\sum_{j=1}^{(T-t_{\mathrm{fy}}^{x})\backslash t}\min\{R_{j}^{x},K_{\max}\cdot (1‐\tau )\}$$ (8)

Here, *P*^*S*^ and *P*^*X*^ is the number of passengers getting on up-train and down-train, respectively [*people*]; *t*^*s*^_*fy*_ and *t*^*X*^_*fy*_ are up-train and down-train departure delays respectively [*s*]; *R*^*s*^_*j*_ and *R*^*X*^_*j*_ represent the number of passengers waiting when up-train and down-train enter Platform 2, respectively [*people*].

### Risk propagation along a metro network

#### Qualitative risk analysis

**Fig 2:**
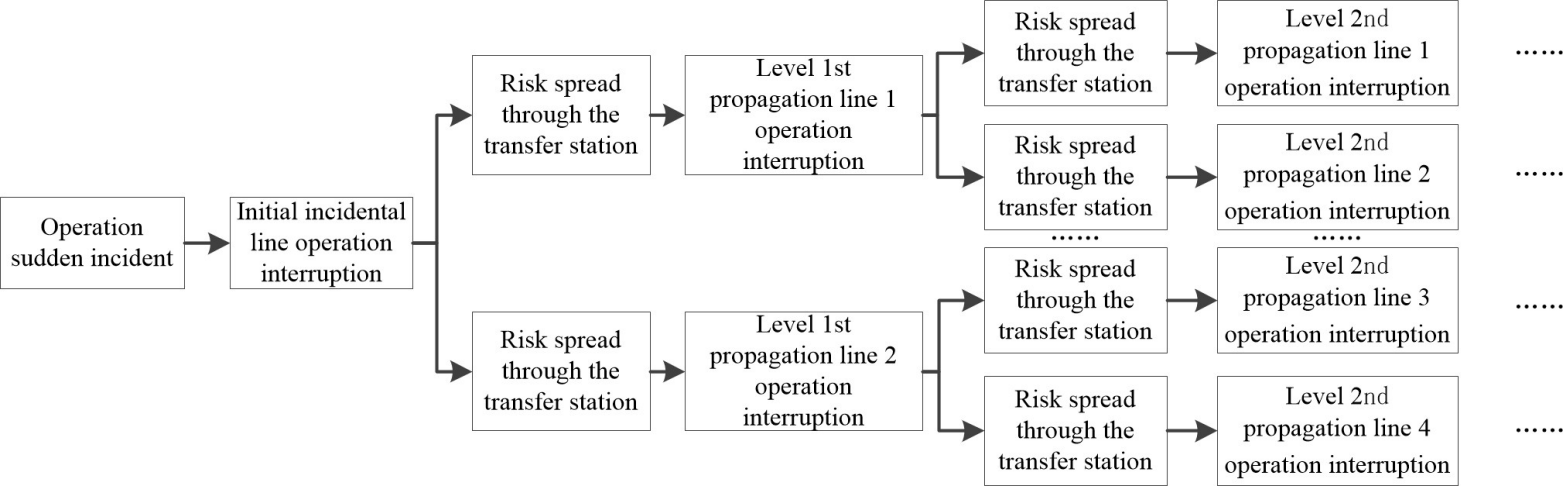
The dimensionality, process, and specific negative effects to the metro operation.

If there is an incident happening on a line in a metro network, the incident risk might propagate to other lines (and perhaps the entire network) through transfer stations. First, there will be passenger retentions in various stations along the line of the incident [37] as stranded passengers in transfer stations will enter the platforms of transfer lines through transfer tunnels, thereby increasing the overcrowding at various platforms. Second, these congestions will spread to other stations through transfer line operations. As the passenger congestion exceeds the maximum transfer platform capacity, the platform could fail, possibly increasing the likelihood of transfer line suspension. Finally, the effects of the incident will probably propagate to the whole network in the absence of effective emergency measures. Overall, the negative effects to the metro network include, but are not limited to, the number of affected lines, the number of trains suspended, and amount of passenger retention at various lines. The dimensionality, process, and specific negative effects to the metro operation are further illustrated in Fig 2.

**Fig 3:**
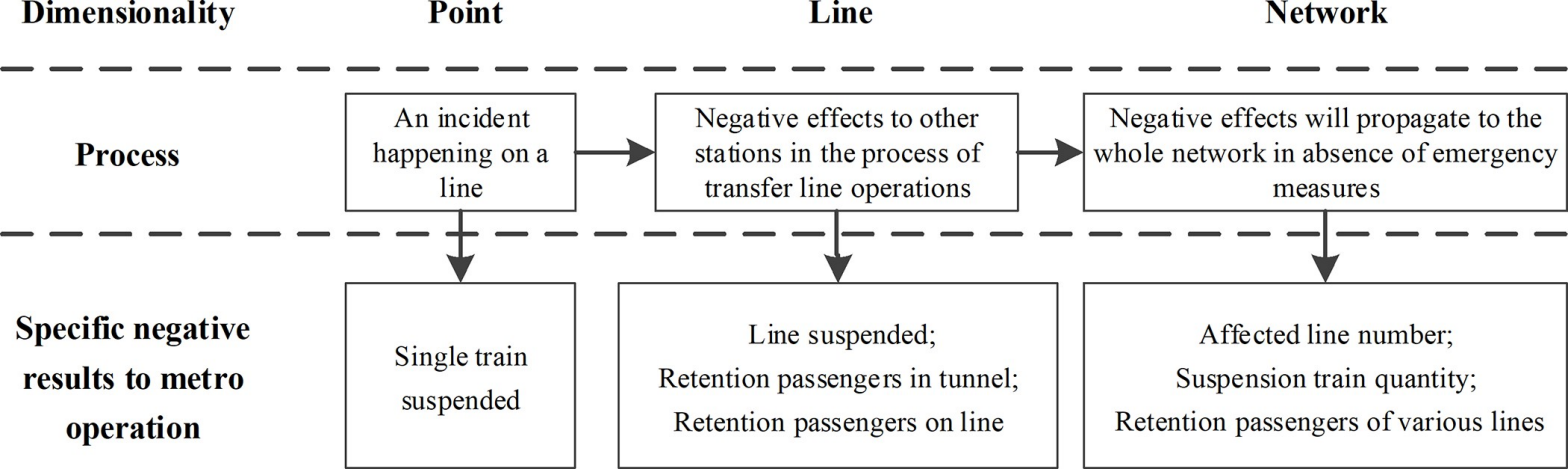
The incidental risk propagation path in a metro network.

The transfer lines that are directly connected to the line associated with the initial incident line by transfer stations are named Level 1 propagation lines. Transfer lines linked to Level 1 propagation lines are defined as Level 2 propagation lines, and so on. Under the condition of the metro network operation, the initial incident line is connected with several Level 1 propagation lines by transfer stations. The incidental risk will spread to Level 1 lines from the initial line, after which the risk will be spreading to Level 2 lines in a similar manner. The incidental risk propagation path in the metro network is displayed in Fig 3.

### Quantitative risk calculation method

Parameters are assumed as follows: (1) all operation parameters on train lines such as train departure interval, driving speed, and station dwelling time are considered to be the same; (2) for all transfer stations, the duration from the retention of passengers from an incidental line entering into transfer station to the number of passengers in the transfer platform reaching maximum capacity, defined as transfer station failure buffer time hereinafter, is deemed to be identical.

#### Duration of risk propagation through the network

(a) ***Single line operation suspension buffer time (T***_***h***_***)***: This value represents the time from when an incident happens on a line to the time when all trains on the line are forced to cease operation and can be calculated by Eq (9):

$$T_{h}=(m-1)\cdot t_{h}$$ (9)

Here, *m* is the number of trains in operation on an incidental line and *t*_*h*_ is the duration of a train coming to a halt.

(b) ***Transfer station failure buffer time (S***_***h***_***)***: As mentioned earlier, when the number of passengers exceeds the capacity of the transfer station platform, the platform can get crowded to the point where trains cannot leave station according to the original schedule, thereby leading to an operation failure of the transfer station and possibly the transfer line. The transfer station failure buffer time *S*_*h*_ is defined as the duration from when passenger retention of an incidental line enters into transfer station to the number of passengers in a transfer platform reaching maximum capacity, which is the duration of average crowding degree at the platform *F*(*T*) reaching to 100%, as shown in Eq (10):

$$S_{h}=\{T|F(T)=1\}$$ (10)

Here, *T* is the incident duration [*s*].

(c) ***Duration of risk propagation along the network (Y)***: In this study, the duration of risk propagation along a metro network is defined as the time from the occurrence of an incident to the whole metro network operation outage, as a result of risk diffusion. It can be computed using Eq (11). Based on Eqs (9–10) and Chen et al. [37], the transfer station failure buffer time *S*_*h*_ is much higher than the suspension buffer time of a single line operation *T*_*h*_. Therefore, risk propagation along the same level lines are not considered.


$$Y=\max\{T^{0}{}_{h}+\sum_{i=1}^{q}T^{i}{}_{h,j}+q\cdot S_{h}|j=1,2,\ldots ,n_{q};S_{h}^{i-1\to i}=S_{h}\}$$ (11)

Here, *T*^*0*^_*h*_ is the operation suspension buffer time of the initial incident line [*s*]; *i* is line level when the incident risk spreads along transfer station, *i* = 1,2,…,*q*; *q* is the final level that the incident risk spreads to through the network originating from the initial line; *T*^*i*^_*h*,*j*_ is the operation suspension buffer time of the *j* line of the *i* level propagation [*s*]; *n*_*q*_ is the line number of *q* level propagation; and*S*_*h*_^*i-1→i*^ is the transfer station failure buffer time when *i*-1 level lines propagating to *i* level lines.

#### Suspension train quantity in the metro network (M)

This number represents the total number of suspended trains across all lines and can be calculated using Eq (12):

$$M=\sum M_{i}$$ (12)

Here, *M*_*i*_ is the number of suspended trains on each line *i*.

#### Retention passenger quantity in the metro network

The retention passenger quantity is determined by the sum of the number of passengers in the metro network at that time.

## Simulation results

Regarding risk propagation along transfer station, risk variance of the side platform only was simulated because the results of the island platforms are similar to those of side platforms. The model equations were employed using the AnyLogic 7.2, a simulation software capable of conducting system dynamics simulation including continuous and real-time scenarios, similar to the context of this study.

### Case study and baseline model development

**Fig 4:**
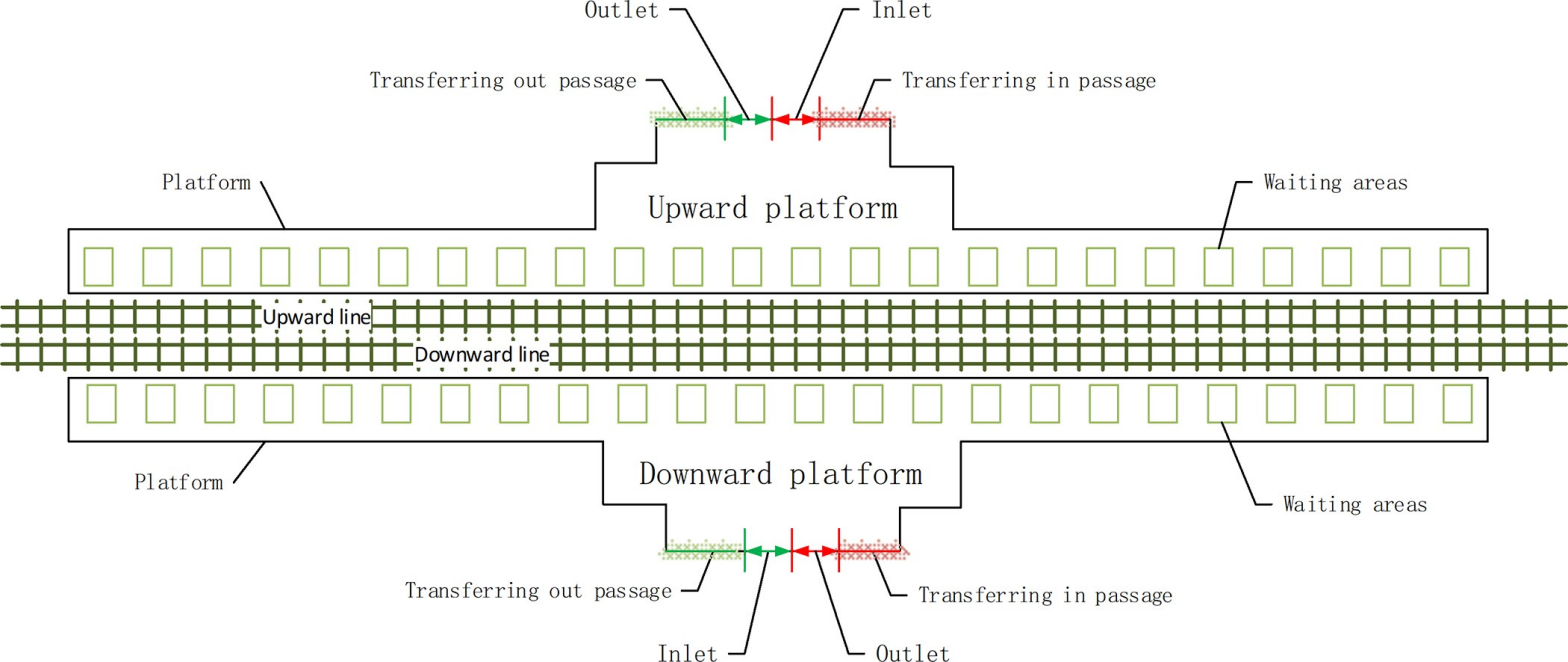
Transfer station side platform simulation structure.

A transfer station side platform is supposed to be 120 meters long and 5 meters wide, as illustrated by the Huixinxijienankou station of Line 10 in Beijing (as shown in Fig 4). The up and down direction trajectories are in the middle of the platform. On both sides of the trajectories reside the platforms. At this station, there are inlets and outlets. There are also two passages for transferring in (green lines) and out (red lines). Also, the grey frame represents the waiting areas near the train doors. The braces and rails in the platform are not considered.

The simulation model parameters are as follows:

1. Simulation time: Simulation time is considered to be 1 rush hour beginning from a departure of Line 2 train.
2. Trains: Trains from each direction enter the station simultaneously. Because each train entering the station is fully loaded, percentage of passenger flow *τ* increases from the normal 60% to 80% and 70% of passengers getting off will decide to get on the train going on the opposite direction to pursue another transfer route as described in Section 2.1.1.
3. Passengers: The number of passengers stranded on Platform 1 is 3,000. They enter Platform 2 through a transfer tunnel for 15 minutes. Moreover, their probability of going in either direction (up or down) is 50%. Meanwhile, the passengers entering one side of Platform 2 coming from the inlets increase to 4000 $\left[\frac{people}{hour}\right]$. The data are assumed based on the passenger flow statistics of the Beijing metro operating company.
4. Other parameters: The parameters associated with the operation of Line 2 and Platform 2 are shown in Table 1.

**Table 1:** Operation parameters of Line 2 and Platform 2.

Parameter	*K*_max_[people]	*t*_td_ [s]	*Z* [people]	*t* [s]	*τ*	*μ*	*γ* [people •s^-1^]
**Value**	1760	20	4000	120	0.8	0.7	2

### Verification

**Fig 5:**
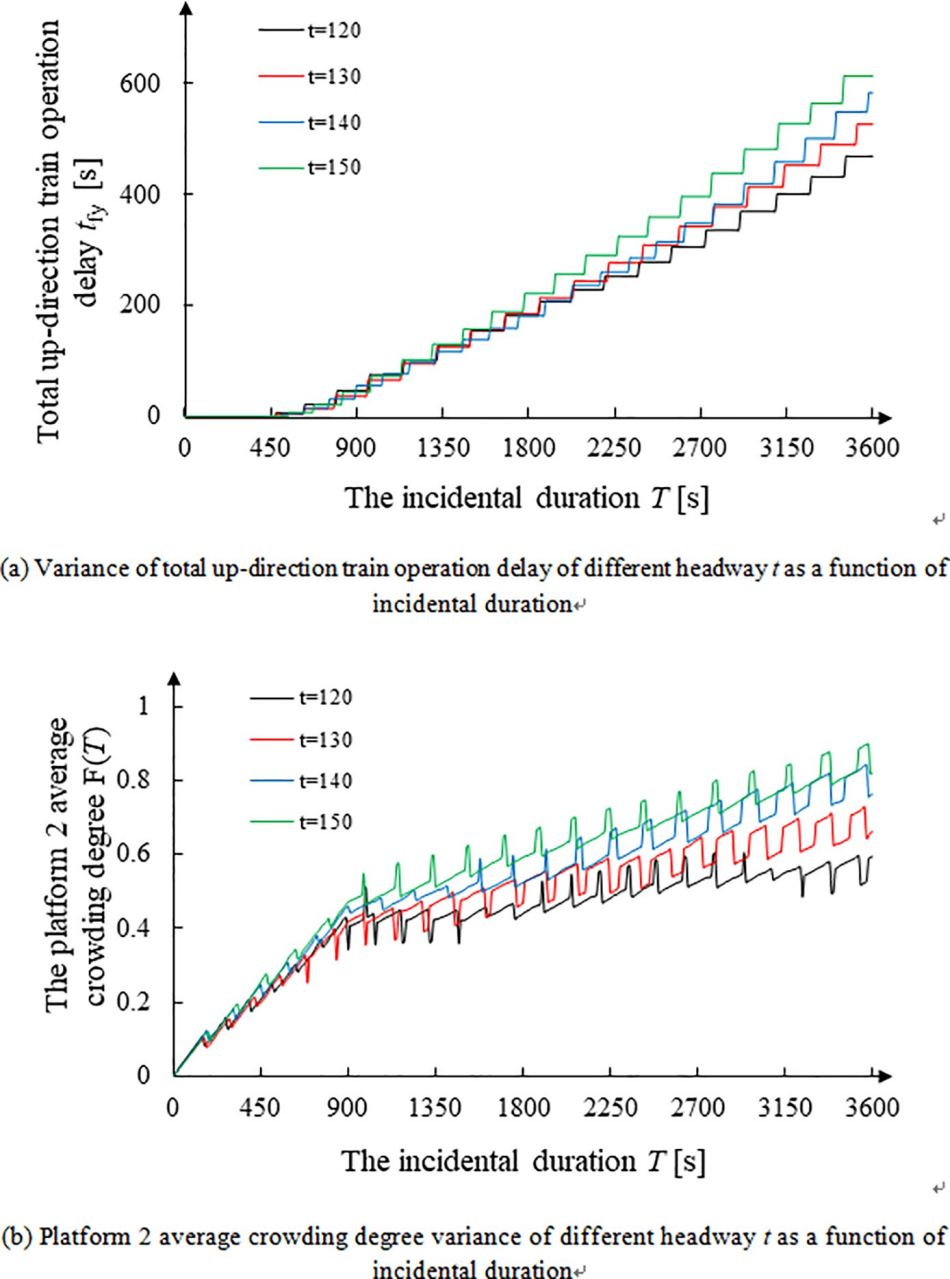
Comparison of simulation results of risk indicators corresponding to different train headway in transfer to Platform 2 (side platform) in the up direction. **(a)** Variance of total up-direction train operation delay of different headway *t* as a function of incidental duration. **(b)** Platform 2 average crowding degree variance of different headway *t* as a function of incidental duration.

Fig 5(A) depicts the variance of total up-direction train operation delay of different headway *t* with incidental duration while Fig 5(B) shows the Platform 2 average crowding degree variance of different headway *t* with incidental duration *T*.

**Fig 6:**
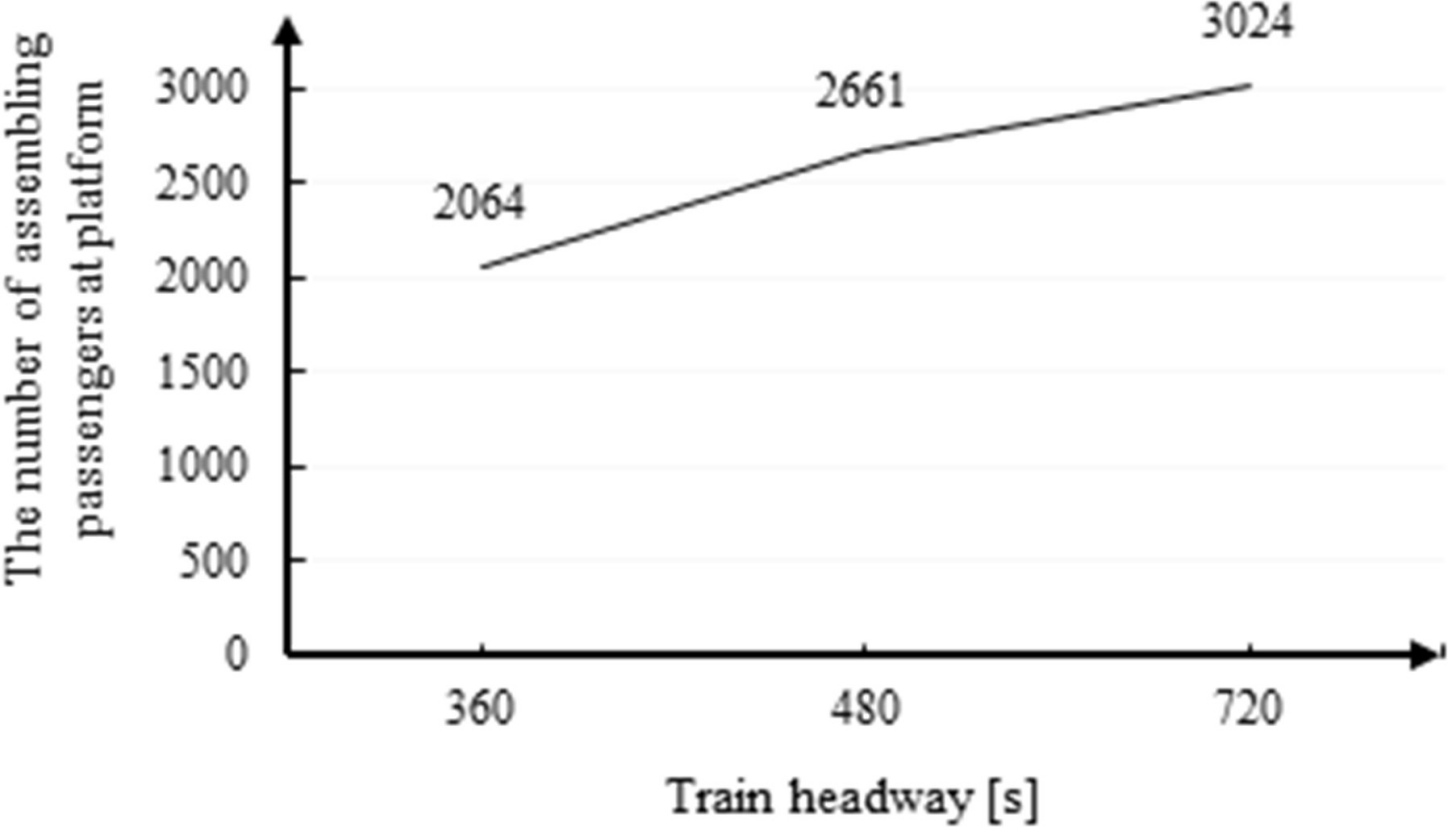
The effect of train headway on the number of assembling passengers at a platform.

Fig 5 shows that the risk indicators, including total train operation delay and average passenger crowding degree on Platform 2, increase as the duration of the incident increases. However, there is little difference among the different impacts of headway *t* for both indicators. Therefore, it can be concluded that the train headway does not have a significant impact on the platform risk indicators. This is because the increase of train headway *t* will reduce the total transportation capability. However, owing to the large number of stranded passengers coming to Platform 2 at the early stage of an incident, the degree of platform crowding is faster in the former phase and slower in the latter phase. In other words, the augmenting extents of headway *t* increase are mild, which are paralleled with the results shown in Fig 6 Aggregating passengers (i.e., the crowding degree) will grow as train headway *t* increases but at a small rate [38].

In addition, Fig 5(B) demonstrates that the curves of the average platform passenger crowding degree are jagged as the number of passengers suddenly increase and decrease as a result of loading and unloading when the train stops at the platform. Zhao et al. [39] observed that in a train operation cycle, the platform aggregated passenger variance rule is increase sharply-to-decrease sharply and then decrease slowly-to-increase slowly. This result is roughly consistent with the cyclic trend of the simulation curve shown in Fig 5(B).

## Discussion

### Variance of risk indicators with passenger-arrival rate

**Fig 7:**
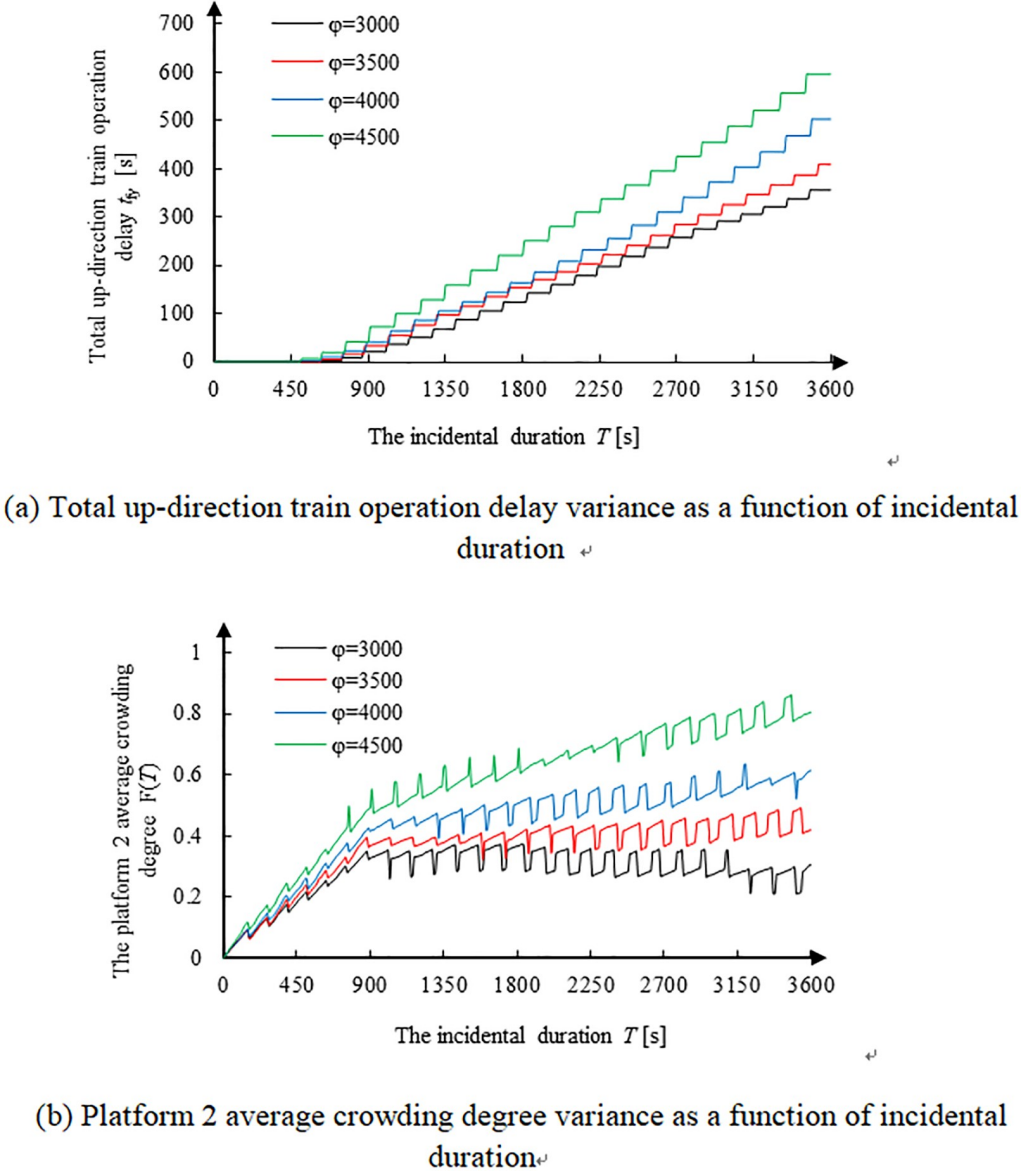
Risk indicators variance of train operation delay and platform crowding degree with passenger-arrival rate *φ* in transfer to Platform 2 (up direction). **(a)** Total up-direction train operation delay variance as a function of incidental duration. **(b)** Platform 2 average crowding degree variance as a function of incidental duration.

Fig 7 shows the risk indicators variance of train operation delay and platform crowding degree with passenger arrival rate *φ*. While Fig 7(A) shows the total up-direction train operation delay variance with incidental duration, Fig 7(B) shows Platform 2 average crowding degree variance. Herein, the passenger arrival rate *φ* is considered for passengers entering the platform through the inlet only. Those entering through transfer tunnels were excluded.

Fig 7(A) shows that train operation delay increases with incidental duration. In addition, the number of passengers coming from platform inlets increases (*φ* value is higher) at a higher rate. On the other hand, Fig 7(B) shows that before 900s the number of passengers and degree of crowding increase quickly, because the retention passenger swarm into Platform 2 sharply and train capacity is less. During the period of 900–1,100s, the crowding degree seems to slow down. After 1100s, all the retention passengers have arrived at Platform 2 and the passenger arrival rate decreases. However, so many significant interaction of passenger flows is observed, thereby reducing loading and unloading efficiency, further slowing down the speed of passengers departing the platform. Afterward, passenger crowding degree increases slowly.

Specifically, the crowding degree of up-direction Platform 2 tends to increase when *φ*≥3500, primarily due to the fact that the passenger arrival rate is more than the train maximum capacity. Consequently, the retention of passengers on Platform 2 increases. On the other hand, this value decreases and the speed of decline becomes faster when *φ*<3500 and *T*≥1800s. This can be attributed to the fact that the passenger arrival rate is less than train maximum capacity and passengers in Platform 2 are inclined to drop off. Hence, the single train operation delay of Line 2 will decrease, and the total passenger transportation capacity rises. As a result, the decreasing speed of the platform crowding degree accelerates.

### Comparison of risk indicators of propagation among metro networks

**Fig 8:**
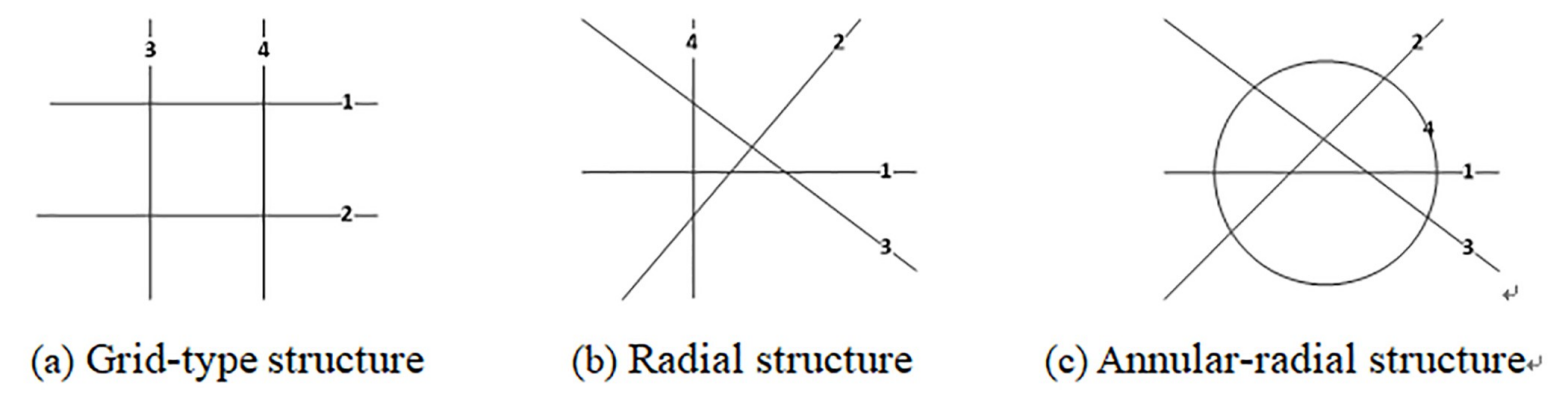
Different structure of metro network. (a) Grid-type structure. (b) Radial structure. (c) Annular-radial structure.

As far as various structures of metro networks are concerned, their risk propagating process and rules are different as a result of network connecting rate discrepancy. To compare the incidental risk propagating rules of various networks, three typical networks are introduced as shown in Fig 8: a) grid, b) radial, and c) annular-radial structure. All networks have 4 lines, and their operation parameters are illustrated in Table 2.

**Table 2:** Operation parameters of lines on typical metro structures.

Parameter	Train quantity of single-direction line				Single train buffer time [s]	Transfer station failure buffer time [s]
	1	2	3	4		
**Value**	30	40	48	54	30	2400

**Fig 9:**
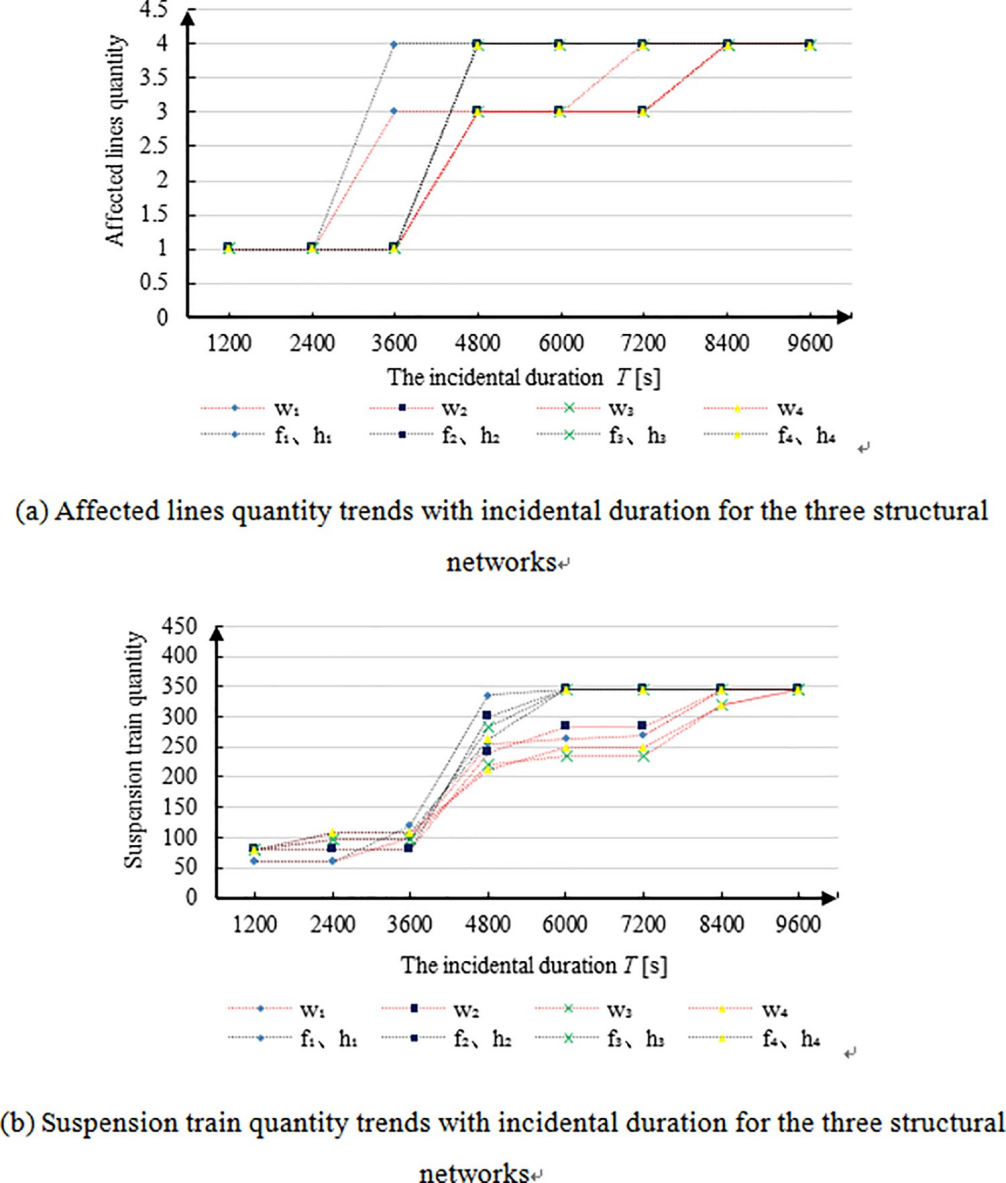
Metro operation risk indicator change trends with incidental duration for the three structural networks. **(a)** Affected lines quantity trends with incidental duration for the three structural networks. **(b)** Suspension train quantity trends with incidental duration for the three structural networks.

Fig 9 displays the metro operation risk indicator change trends with incidental duration for the three structural networks. Fig 9(A) shows the affected line quantity with respect to the incidental duration. Fig 9(B) shows the suspension train quantity. For the sake of finding rules, an imaginary line was used to connect the discrete values of the results. In Fig 9, “*w*” represents the grid-type network, “*f*” represents the radial network, and “*h*” refers to the annular-radial network. Besides, the numbers “1–4” indicate Lines 1 to 4, respectively.

For a grid-type network, as shown in Fig 9(A), we can see that two risk propagations through transfer stations are required for all lines to be suspended when the four lines are considered as initiating incidental lines, one at a time, and w_2_, w_3_, and w_4_ curves are identical as lines 2, 3, and 4 being the incidental line in order. For radial networks, only one risk propagation through transfer stations is needed for all lines suspension, also f_2_, f_3_, and f_4_ curves are identical as line 2, 3, and 4 are being the initial lines orderly. Moreover, the affected line quantity trends of 4 lines as initiating incidental line for the annular-radial network are totally consistent with those for the radial network; therefore, f_1_ and h_1_ are represented by only one line and f_2_ and h_2_ are indicated by one line, and so on.

As can be seen from Fig 9(B), the suspension train quantity trends of the radial network as line 1–4 being the incidental lines one at a time are the same as those of the annular-radial network. Specifically, f_1_ and h_1_, f_2_ and h_2_, f_3_ and h_3_, and f_4_ and h_4_ coincide, respectively. Therefore, we used one line to represent the two networks as described above. All values on the curves increase rapidly during the period of 3600-4800s. The curves of the radial and annular-radial networks increase faster than those of the grid-type network. Moreover, after 4800s, the curve of the grid-type network increases slowly until the final state, when all lines are suspended, is reached.

It can be inferred that the risk indicators (including the affected line quantity and suspension train quantity) propagation speed of the grid-type network is relatively slow. In a grid, there are parallel lines whose connecting degree is relatively slow, and the incidental risk effect needs more time to propagate to the transfer station when all lines are suspended. Furthermore, grid-based networks resist destruction better than the other structures, but their passenger service efficiency is lower as more transfers are needed to arrive at a destination. On the hand, the risk propagation speed of the radial and annular-radial networks is relatively faster, because two arbitrary lines, in these networks, are mutually connected so that the incidental risk could spread to the whole network through only one transfer station propagation. The damage resistance ability of these two networks is relatively worse. However, the passenger service efficiency is higher because fewer transfers are needed to reach a destination.

Concerning the condition that there is more than one transfer station between two lines, just one transfer station propagation is considered, thus causing the risk indicators curves of the radial and annular-radial networks to be consistent. In practice, as for annular-radial network, there is more than one transfer station between the annular lines and other lines, thus leading to faster risk propagation through all of the network. In summary, the order of three networks, from high to low, in terms of the incidental risk propagation speed is as follows: annular-radial, radial, and grid-type.

## Conclusion and limitations

Rules of operation incidental risk propagation in a metro network under fully automatic operation mode were explored. The indicator computation models for transfer station and different structural networks were developed and indicator variance rules were discussed and verified through a simulation study. The computational models were applied and the network risk propagation rules were obtained. A summary of the conclusions from this study are presented as follows:

1. During the incidental risk propagation along a metro transfer station, there is a great effect on non-incidental line platform operation as detained passengers suddenly influx into the platform. Specifically, a train operation delay occurred at 450s for non-incident lines. Moreover, when passengers coming through the platform inlets are more, the faster reaching the non-incidental line platform total train operation delay and the higher the crowding degree. However, train headway discrepancy has little influence on non-incidental line platform risk development.
2. Regarding the incidental risk propagation through a metro network, the propagation speed varies for differently structured networks. More specifically, the propagation speed of annular-radial network is the fastest, with the radial network being slower and the grid network being the slowest. Generally, the longer the incidental line in network is (i.e., more operation trains on the line), the longer the duration of risk propagation along the network. Conversely, the shorter the incidental line in network is (i.e., fewer operation trains on the line), the shorter the duration of the risk propagation along the network.

The conclusions as above are supposed to be supports for metro operation safety planning and network design. Comparing to traditional operation mode, the fully automatic operation mode applies CBTC (Communication Based Train Control) system and it can reduce train headway and improve transport efficiency greatly. However, it will give greater challenges for operation team when an emergency occurs, since it could affect more trains and passengers in the same period than those under traditional operation mode. In this paper, emergency dispatching measures such as closing transfer channel and withdrawing train was not taken into account. The research considers the worst-case scenario that the staff fails to take emergency measures after an incident occurred. Thus the results of risk assessment can guide the metro operation planning and network design under traditional operation mode to the most conservative extent.

## Supporting information

S1 File (ZIP)
