# Supplementary material for: Rules of incidental operation risk propagation in metro networks under fully automatic operations mode
Source: PLoS One. 2021 Dec 16;16(12):e0261436. doi: 10.1371/journal.pone.0261436 (PMC8675654; doi:10.1371/journal.pone.0261436)
Supplement: S1 File — (ZIP) [file pone.0261436.s001.zip › 1Manuscript.docx]

***Rules of incidental operation risk propagation in metro networks under fully automatic operations mode***

Wenying Chen1,2,*, Jinyu Yang1,#a,2, Mohammad T. Khasawneh3,#b, Jiaping Fu1,2，Baoping Sun1,2

1 School of Management and Engineering, Capital University of Economics & Business

2 Beijing Key Laboratory of Megaregions Sustainable Development Modeling, China

3 Department of Systems Science and Industrial Engineering, State University of New York at Binghamton

#a Current Address: School of Management and Engineering, Capital University of Economics & Business, Flower-Town, Fengtai District, Beijing 100070, China

#b Current Address: Department of Systems Science and Industrial Engineering, State University of New York at Binghamton, Binghamton, NY 13902, USA

* Corresponding author

E-mail: chenwy@cueb.edu.cn (WC)

**Abstract**

The frequent interruptions of network operation due to any incident suggest the necessity to study the rules of operational risk propagation in metro networks, especially under fully automatic operations mode. In this study, a point-to-line-to-network scheme was adopted and risk indicators were determined by analyzing risk propagation processes within transfer stations and metro networks. This research develops mathematical-based network models to understand risk propagation in metro networks. Moreover, indicator variance rules for a transfer station and different structural networks were discussed and verified through simulation. After reviewing the simulation results, it was concluded that under the impacts of both sudden incident and peak passenger flow, the more the passengers coming from platform inlets, the longer the non-incidental line platform total train operation delay and the higher the crowding degree. However, train headway has little influence on non-incidental line platform risk development. With respect to incident risk propagation in a metro network, the propagation speed varies with network structure, wherein an annular-radial network is the fastest, a radial is moderately fast, and a grid-type network is the slowest. The conclusions are supposed to be supports for metro operation safety planning and network design.

***Keywords*:** Operation incident; Incident duration; Network structure; Risk propagation rule; Risk indicator

**Introduction**

Rapid urbanization in China has greatly boosted the initiation of increased metro rail transit projects throughout the nation. By 2017, 34 cities had 165 rail transit lines in service of which the total length is 5033 km [1]. However, along with service on numerous metro lines come not only the benefits of road traffic relief and environmental pollution reduction but also new challenges and demands for reliable and safe metro operation [2]. When it comes to network operation, the relations among different lines and stations are highly complex, wherein an incidental risk could be propagated in the entire rail transit network as a result of a local fault or a failure. Once an incident leads to local failure, the burden from passengers on other parts of the railway increase immediately, thereby disabling the whole network owing to behaviors such as overloading. If a local incident cannot be addressed in a timely manner, it will lead to negative effects such as passenger retention, train delay, and cessation of metro line service, and potentially even more serious casualties. For example, on November 22, 2009, a crash occurred on Metro Line 1 in Shanghai because of equipment failure. In addition to anxiety among passengers, this failure caused all the metro lines to fail for five hours. Another example is that a mass of train delays happened on Metro Line 10 in Beijing owing to a signal fault, leading to the cessation of 101 trains and disturbing more than200,000 people [3]. Therefore, rules of incident operation risk propagation through metro networks are needed to study the negative effects of incidents on metro lines and trains in such complex networks.

With the rapid development of Artificial Intelligence (AI), fully automatic operations have become prevalent. Such operational mode has been already adopted for Shanghai Metro Line 10, the Beijing Subway Line Yanfang, and several other lines in China. Compared to traditional modes of operation, the operation safety risk arising from human behavior in automated modes could be reduced and operational efficiency can be improved. This can be attributed to the full-automatic functions of line operation, in and out of the station, that can be accomplished; functions such as door opening and closing and shutoff and initiation, etc. However, emergency response capacity might be impaired under this model. When a train in the line has to be shut off as a result of an incident, such as faulty equipment, following trains will have to apply brakes to cease successively dominated by Automatic Train Protection (ATP). If the faulty train cannot be removed in time, other trains on the line will be suspended for a period. The incident risk will spread to other lines through transfer stations leading to a cessation of the entire network operation.

Several studies discussed safety analysis for general subway operations such as Xu et al.[4], and Ahmad and Khan [5]. Kyriakidi et al. [6] analyzed accident data from 2002 to 2009 and developed a comprehensive assessment using a safety maturity model for the metro operation, which considers not only human behaviors and attitudes, but also the technical and operational factors. Louie et al. [7] focused on the effects of the incidents’ location and occurrence time, the train-type involved, and the non-adherence to proper recovery procedures in an effort to investigate the relationship between those factors and the resulting delay duration using the Accelerated Failure Time (AFT) hazard model. Zhang et al. [8] introduced details of all incidents that have occurred in metro operations toprevent future accidents using an adaptable metro operation incident database. Also, Lu et al. [9] introduced a metro operation risks framework that incorporates accident causes, processes, and solutions to prevent accidents using a new case-based reasoning method. Given a similar baseline, the safety risk and safety measures of an input case can be analyzed qualitatively based on the retrieved case. Chen et al. [10] presented the causal chains of Beijing metro operation risks initiated from a human factors perspective and discussed the uncertain logical relationship between events in the chain and the possibility fluctuation of event occurrences.

In spite of the safety analysis of subway operations as discussed above, the current research on metro network risk propagation focuses on two aspects: 1) metro network reliability and [destroy-resistant ability](https://kns-cnki-net.webvpn.cueb.edu.cn/kcms/detail/knetsearch.aspx?dbcode=CJFD&sfield=kw&skey=destroy-resistant%20ability&code=&uid=WEEvREcwSlJHSldRa1Fhb09jT0pjWkNMcmNraDFreVRhNDI5K3J5R2xSQT0=$9A4hF_YAuvQ5obgVAqNKPCYcEjKensW4IQMovwHtwkF4VYPoHbKxJw!!) and 2) sudden massive passenger flow and congestion propagation.

As far as metro network reliability is concerned, there is very limited research in this area. The concept of transportation network connectivity reliability is based on maintaining the connectivity probability between two points in a transportation network, which was issued originally by Mine and Kawai [11]. Iida et al. [12] used a computation method of minimal path set and cut set for road network terminal reliability and applied the shortest path computation method for network connectivity reliability. On the other hand, Sanso et al. [13] used a minimal cut set method for calculating transportation network connectivity reliability to solve the cyclic problem of route selection. Bell and Iida [14] first described the definition and calculation method in detail in the book “Transportation Network Analysis”. Latora et al. [15] discussed the center point of a metro network by studying the Boston metro and came up with the concepts of global efficiency and component efficiency. Crucitti et al. [16] showed classical network connectivity variance after random and deliberate attacks by evaluating network efficiency, proving that their network efficiency evaluation method is superior to others. Han et al. [17] studied the reliability of the Shanghai Metro network by computing relative scales of the maximal connected subgraph, global efficiency and component efficiency indexes. Luo et al. [18] employed the Pajek method using MATLAB to study the reliability of the Shenzhen Metro network. They found that when facing deliberate attacks, key nodes of the network have a greater effect on the efficiency of the entire network.

A few attempts have been made to study metro network [destroy-resistant ability](https://kns-cnki-net.webvpn.cueb.edu.cn/kcms/detail/knetsearch.aspx?dbcode=CJFD&sfield=kw&skey=destroy-resistant%20ability&code=&uid=WEEvREcwSlJHSldRa1Fhb09jT0pjWkNMcmNraDFreVRhNDI5K3J5R2xSQT0=$9A4hF_YAuvQ5obgVAqNKPCYcEjKensW4IQMovwHtwkF4VYPoHbKxJw!!). Angeloudi and Fisk D. [19] found that a metro networkhavehigher survivability against a random attack compared to relatively lower survivability in the case of adeliberate attack based on the node importance degree. Wang et al. [20] presented a vulnerability assessment method of urban metro network topological structure and concluded that the vulnerability is apparent when a metro station is attacked deliberately while the robustness is obvious when station-to-station tunnels are assaulted in the same manner. Yang et al. [21] revealed that the Beijing subway system exhibits typical characteristics of a scale-free network, with relatively high survivability and robustness when faced with random failures, whereas, based on complex network theory, error tolerance is relatively low when the hubs undergo malicious attacks. Diao [22] defined metro network [destroy-resistant ability](https://kns-cnki-net.webvpn.cueb.edu.cn/kcms/detail/knetsearch.aspx?dbcode=CJFD&sfield=kw&skey=destroy-resistant%20ability&code=&uid=WEEvREcwSlJHSldRa1Fhb09jT0pjWkNMcmNraDFreVRhNDI5K3J5R2xSQT0=$9A4hF_YAuvQ5obgVAqNKPCYcEjKensW4IQMovwHtwkF4VYPoHbKxJw!!) as the ability of maintain the metro network passenger transportation efficiency after self-malfunction or destruction from outside under certain circumstances and within a specific period.

Wu et al. [23] introduced the tolerability index measuring the vulnerability of metro systems from the perspective of passengers, which is generated as a combination of satisfying route, shortest path, and weighted average. This new index will help managers identify the most important stations and allocate resources efficiently and effectively to enhance their protective capability. Jenelius et al. [24] focused on the importance of network edge and site in vulnerability analysis, thinking that the vulnerability is mainly composed of the probability of component failure and its consequences. Moreover, the Beijing Subway network vulnerability was compared with Shanghai’s and it was concluded that the more complex a metro network structure, the lower its vulnerability [25]. Xu et al. [27] combined the metro accidental spreading mechanism with a gravity model to construct a model on the foundation of justifying network vulnerability as an accidental spreading characteristic parameter. Their model was verified by Beijing metro network.

Overall, the literature discusses the network transportation efficiency and connectivity after a station or station-to-station tunnel is attacked randomly and deliberately. These studies examined just the static topological structure of a metro network. They did not consider the dynamic effects of a risk carrier (i.e., passenger flow) in the metro risk propagation process.

Because of the confined underground space, problems related to passenger flows and their developments have been widely addressed in the literature. Assis et al. [27] discussed the characteristics of passenger flow dynamic variance over time. Castelli et al. [28] developed a method for formulating train timetables that considered the variance of passenger flow with time and space of all metro lines connected by atransfer station. Garcia and Martin [29] developed a network equilibrium model that allocated passenger flow for different lines in a metro network given different travel demands. Silva et al. [20] studied the effects of a station closure or interruption, as a result of a sudden accident, on passenger flow behavior and station crowdedness. Chen et al. [31] applied the Susceptible Infected Recovered Model (SIRM) to discuss the rules of massive passenger flow propagation in a metro network. Gao et al. [32] studied the number of passengers stranded at a station because of faulty subway operations and proposed an optimized, iterative algorithm based on the Beijing subway network, by rescheduling the train stop plan to alleviate overcrowding problems. Jiao [33] developed a passenger congestion propagation model based on Cellular Automata (CA) and SIRM and proposed a set ofpassenger congestion propagation rules for the Beijing metro network. Zheng [34] developed a system dynamic model for metro passenger flow and presented a [synergistic](file:///C:\Users\chenwy\AppData\Local\youdao\dict\Application\7.2.0.0703\resultui\dict\?keyword=synergistic) control method for passenger flow based on a congestion propagation model. Huang et al. [35] studied the temporal-spatial erosion process through a congestion propagation model and identified various control points. Xiao et al. [36] developed a dynamic passenger congestion propagation model to account for the dynamics of disaster spread.

The studies above focused on aggregation and congestion propagation rules of commuting passenger flow or passenger flow caused byalarge-scale activity, whereas few researchers have explored passenger flow propagation induced by a sudden incident. In addition, little research has been done on operational risk and passenger flow propagation in a metro network caused by a sudden incident and peak passenger flow simultaneously.

Nevertheless, the effects of an incident will spread through an operating metro network under various conditions and operational modes. First, the incidental line will be suspended or be forced to modify the operation program because of the possibility of technical limitations. On the other hand, incidental risk will spread to other lines through transfer stations, whose carrier is “passenger flow”. Consequently, this will lead to a huge burden on passenger organizations for these lines. Specifically, when rush hour passenger flow overlaps with incident-caused congestion, the negative consequences on the network will result in a “snowball effect,” slowing down trains and increasing station crowding, in addition to other serious secondary accidents such as a stampede. In conclusion, sudden incident risk propagation in a metro network is highly dependent on metro line topological structures, passenger flow variance, and operation dispatching measures. Therefore, in this research, sudden incidental risk propagation mechanism and rules among metro networks are discussed considering both static network topological structure and dynamic passenger flow.

This study followsapoint-line-network order starting from an incident or an emergency in a single line. For a single line, the relationship between increase speed of stopped trains and line occupancy rates as a function of incident duration, train headway and stopped trains have been studied by Chen et al. [37] . In this research, on the other hand, the incident risk and peak passenger flow propagation along transfer stations and consequently other lines, under full-automatic operation mode, are emphasized.

The rest of this article is organized as follows. Section 2 presents a qualitative analysis of risk propagation through ametro network and a quantitative computational model of risk indicators. Section 3 discusses risk indicator variance at a transfer station’s side platform caused by an incident and how risk propagation indicators vary among metro networks. Finally, Section 4 summarizes and concludes this paper.

**Method**

**Risk propagation along transfer station**

**Qualitative risk analysis**

In this study, a train entering an island transfer station during rush hour is used as an example for the qualitative risk analysis and quantitative risk evaluation in cases of failures due to an incident in the metro network. For convenience, the incident line is called Line 1, while the other line connecting with Line 1 by the transfer station is called Line 2. The platform of transfer station linked by Line 1 is Platform 1 and that linked by Line 2 is Platform 2.

Fig 1 shows the incidental risk propagation paths along a transfer station. When Line 1 is suspended due to an incident, there are several changes of passenger flow in Platform 2: (1) all passenger stranded in Platform 1 will swarm into Platform 2 in a short period of time; (2) passenger planning to come into Platform 1 might change their minds and enter Platform 2, thereby increasing the passengers number at Platform 2; (3) some passengers planning to transfer to Line 1 might choose the opposite direction of Line 2 to pursue another transfer route (therefore, different direction flow of passenger is crossed and the number of passengers in Platform 2 increase); and (4) some passengers on Line 2 who had been planning to transfer to Line 1 might select to go to the next transfer station to pursue another travel route instead of getting off the train as originally planned.

**Fig 1. Risk propagation path of incidental operation along transfer station**

After a fully loaded train comes to a full stop at Platform 2, its residual capacity is decreased because fewer passengers get off. In addition, unloading-loading efficiency is reduced, and stopping time at the platform increases because of the large number of passengers and the lack of order. If the stopping time at the platform is longer than the scheduled time, a train delay takes place.

As the train leaving Platform 2 is overloaded, its available capacity will be less upon arrival at the next station, thereby increasing the overall number of passengers waiting for a train at subsequent stations. Therefore, the number of delayed passengers will increase over time, leading to congestion at other stations along Line 2. Meanwhile, when the train enters subsequent transfer stations of Line 2, there will be passenger overload in these stations because they have to accept additional passengers who were supposed to have gotten off at Platform 2 of the original transfer station. Consequently, the incident risk might propagate into other lines in the network through these transfer stations connected with Line 2. In fact, this could even impact the operation of the entire network in this highly connected system.

In conclusion, the primary indexes of risk propagation along transfer stations are dependent on the operational delay of Line 2 trains passing the transfer station as well as the average crowding degree of the transfer station.

**Quantitative risk computation model**

To quantify the risk propagation, several assumptions have to be made: (1) all trains in the metro network operate automatically; (2) to obtain the rules of risk propagation, train headway, the dwelling time fixed in the train diagram, and passenger flow-through rate, are assumed to be the same for all trains; (3) before the start of the computation process, there are no residual passengers on the train platform as trains depart the station; and (4) trains getting to and departing from a station are at maximum passenger capacity in peak hours.

**Train operation delay**

(a) ***Train departure delays of Line 2*** (): The operation delays of Line 2 up-trains are computed only because those of down-trains are the same. After Line 1 suspension, the Line 2 up-train arriving at Platform 2 is (). The operation delay for a single train is the difference between the actual dwelling time and the dwelling time fixed in the schedule as illustrated in Eq. (1). The actual dwelling time represents the duration through which the train stops at a platform to allow for the loading and unloading of passengers. If the value is less than or equal to 0, there is no delay. The total operation delay of Line 2 up-trains is the sum of each train departure delays as shown in Eq. (2).

(1)

(2)

Here, is total operation delays of Line 2 up-train, (s); is train departure delay at transfer station, (s); is the waiting passenger quantity when train enters Platform 2, is the train passenger capacity, is percentage of passenger flow, is velocity of passenger getting on train per unit time []；and is the dwelling time fixed in the train schedule .

(b) ***Waiting passenger quantity when train entering Platform 2*** (): When train enters Platform 2, the initial waiting passenger quantity *R1*is the number of arriving passengers in a train headway. On the other hand, the number of passengers waiting (*Rj)* when train enters Platform 2 is the sum of the residual passengers on Platform 2 when train departs and the arriving passengers accumulating since train leaves and train enters. The number of residual passengers when train departs is the sum of the number of passengers waiting when train enters () and the number of passengers transferring from the opposite direction train when is at the station, that is (*μ* is the proportion of reversing passengers relative to the quantity of getting off from the opposite direction train), minus the number of passengers getting on train . If the result is less than or equal to 0, the number of residual passengers when train departs is 0. This can be represented in Eqs. (3-4).

(3)

(4)

Here, is the initial waiting passenger quantity ; is the waiting passenger quantitywhen train enters Platform 2 ; is the formal train headway ; is the average velocity of arrival at the platform .

(c) ***Velocity of passengers getting on train*** (): The velocity of passengers getting on a train is determined by the train door number, average velocity of passengers getting on one single door ( ), and cross-influence coefficient of passenger flow on and off the train (). Generally, in Beijing, there are six coaches in a train and four doors per coach. Therefore, there are a total of twenty-four doors per train. The velocity of passengers is represented mathematically as shown in Eq. (5).

(5)

(d) ***Cross-influence coefficient of passenger flow on and off train*** (): The value of represents the loading efficiency due to the number of passengers getting on and off the train and is primary determined by the passenger density near a train door. While passenger density is relatively low, there is no mutual interference. Hence, the duration of unloading (former) and loading (latter) are the same and is 0.5. In contrast, while passenger density is too high, even exceeding the minimum limit of 0.33 [according to China Code for Design of Metro [38], that is, more than 3 [, the mutual interference is very severe. In this case, is proportional to the reciprocal of the square of passenger density [39]. Therefore, the cross-influence coefficient of passenger flow on and off a train () is calculated using Eq. (6).

(6)

Here, is the passenger density .

**Average crowding degree at platform 2 F (T)**

1. ***Average crowding degree at Platform 2*** (*F*(*T*)): According to Li et al. [40], is the ratio of the actual number of passengers waiting and the capacity of the platform. This is illustrated using Eq. (7).

(7)

Here, is the average velocity of people arriving at the platform ; is incident duration ; is the initial number of passengers waiting ; *P* is the number of people departing when the train leaves Platform 2 ; and is the platform passenger capacity .

(b) ***The number of passengers departing when train leaves* (***P): P* is the number of passengers departing when the train leaves Platform 2, including passengers getting on up-train and down-train as captured by Eq. (8).

（8）

Here, and is the number of passengers getting on up-train and down-train, respectively ; and are up-train and down-train departure delays respectively ; and represent the number of passengers waiting when up-train and down-train enter Platform 2, respectively .

**Risk propagation along a metro network**

**Qualitative risk analysis**

If there is an incident happening on a line in a metro network, the incident risk might propagate to other lines (and perhaps the entire network) through transfer stations. First, there will be passenger retentions in various stations along the line of the incident [37] as stranded passengers in transfer stations will enter the platforms of transfer lines through transfer tunnels, thereby increasing the overcrowding at various platforms. Second, these congestions will spread to other stations through transfer line operations. As the passenger congestion exceeds the maximum transfer platform capacity, the platform could fail, possibly increasing the likelihood of transfer line suspension. Finally, the effects of the incident will probably propagate to the whole network in the absence of effective emergency measures. Overall, the negative effects to the metro network include, but are not limited to, the number of affected lines, the number of trains suspended, and amount of passenger retention at various lines. The dimensionality, process, and specific negative effects to the metro operation are further illustrated in Fig 2.

**Fig 2. The dimensionality, process, and specific negative effects to the metro operation**

The transfer lines that are directly connected to the line associated with the initial incident line by transfer stations are named Level 1propagation lines. Transfer lines linked to Level 1 propagation lines are defined as Level 2propagation lines, and so on. Under the condition of the metro network operation, the initial incident line is connected with several Level 1propagation lines by transfer stations. Therefore, the incidental risk will spread to Level 1 lines from the initial line, after which the risk will be spreading to Level 2 lines in a similar manner. The incidental risk propagation path in the metro network is displayed in Fig 3.

**Fig 3. The incidental risk propagation path in a metro network**

**Quantitative risk calculation method**

Before calculating the incidental risk to obtain the propagation rules, some parameters are assumed as follows: (1) all operation parameters on train lines such as train departure interval, driving speed, and station dwelling time are considered to be the same; (2) for all transfer stations, the duration from the retention of passengers from an incidental line entering into transfer station to the number of passengers in the transfer platform reaching maximum capacity, defined as transfer station failure buffer time hereinafter, is deemed to be identical.

**Duration of risk propagation through the network**

(a) ***Single line operation suspension buffer time (Th)***: This value represents the time from when an incident happens on a line to the time when all trains on the line are forced to cease operation and can be calculated by Eq. (9):

(9)

Here, *m* is the number of trains in operation on an incidental line and *th* is the duration of a train coming to a halt.

1. ***Transfer station failure buffer time (Sh)***: As mentioned earlier, when the number of passengers exceeds the capacity of the transfer station platform, the platform can get crowded to the point where trains cannot leave station according to the original schedule, thereby leading to an operation failure of the transfer station and possibly the transfer line. The transfer station failure buffer time *Sh* is defined as the duration from when passenger retention of an incidental line enters into transfer station to the number of passengers in a transfer platform reaching maximum capacity, which is the duration of average crowding degree at the platform *F*(*T*) reaching to 100%, as shown in Eq. (10):

(10)

Here, *T* is the incident duration .

1. ***Duration of risk propagation along the network (Y)***: In this study, the duration of risk propagation along a metro network is defined as the time from the occurrence of an incident to the whole metro network operation outage, as a result of risk diffusion. It can be computed using Eq. (11). Based on Eqs. (9-10) and Chen et al. (2018), the transfer station failure buffer time *Sh* is much higher than the suspension buffer time of a single line operation *Th*. Therefore, risk propagation along the same level lines are not considered.

(11)

Here, is the operation suspension buffer time of the initial incident line ; *i* is line level when the incident risk spreads along transfer station,; is the final level that the incident risk spreads to through the network originating from the initial line; is the operation suspension buffer time of the *j* line of the level propagation ; is the line number of level propagation; and is the transfer station failure buffer time when *i*-1 level lines propagating to *i* level lines.

**Suspension train quantity in the metro network (M)**

This number represents the total number of suspended trains across all lines and can be calculated using Eq. (12):

(12)

Here, *Mi* is the number of suspended trains on each line *i*.

**Retention passenger quantity in the metro network**

When the lines in the metro network are suspended, all passengers will be stranded. Hence, the retention passenger quantity is determined by the sum of the number of passengers in the metro network at that time.

**Simulation results**

Regarding risk propagation along transfer station, risk variance of the side platform only was simulated because the results of the island platforms are similar to those of side platforms. The model equations were employed using the AnyLogic 7.2, a simulation software capable of conducting system dynamics simulation including continuous and real-time scenarios, similar to the context of this study.

**Case study and baseline model development**

A transfer station side platform is supposed to be 120 meters long and 5 meters wide, as illustrated by the Huixinxijienankou station of Line 10 in Beijing (as shown in Fig 4). The up and down direction trajectories are in the middle of the platform. On both sides of the trajectories reside the platforms. At this station, there are inlets and outlets. There are also two passages for transferring in (green lines) and out (red lines). Also, the grey frame represents the waiting areas near the train doors. The braces and rails in the platform are not considered.

**Fig 4. Transfer station side platform simulation structure**

The simulation model parameters are as follows:

1. Simulation time: Simulation time is considered to be 1 rush hour beginning from a departure of Line 2 train.
2. Trains: Trains from each direction enter the station simultaneously. Because each train entering the station is fully loaded, percentage of passenger flow increases from the normal 60% to 80% and 70% of passengers getting off will decide to get on the train going on the opposite direction to pursue another transfer route as described in Section 2.1.1.
3. Passengers: The number of passengers stranded on Platform 1 is 3,000. They enter Platform 2 through a transfer tunnel for 15 minutes. Moreover, their probability of going in either direction (up or down) is 50%. Meanwhile, the passengers entering one side of Platform 2 coming from the inlets increase to 4000 . The data are assumed based on the passenger flow statistics of the Beijing metro operating company.
4. Other parameters: The parameters associated with the operation of Line 2 and Platform 2 are shown in Table 1.

**Table 1. Operation parameters of Line 2 and Platform 2**

| Parameter | [people] | [s] | [people] | [s] |  |  | [people •s-1] |
| --- | --- | --- | --- | --- | --- | --- | --- |
| Value | 1760 | 20 | 4000 | 120 | 0.8 | 0.7 | 2 |

**Verification**

Fig 5(a) depicts the variance of total up-direction train operation delay of different headway *t* with incidental duration while Fig 5(b) shows the Platform 2 average crowding degree variance of different headway *t* with incidental duration *T*.

**(a) Variance of total up-direction train operation delay of different headway *t* as a function of incidental duration**

**(b) Platform 2 average crowding degree variance of different headway *t* as a function of incidental duration**

**Fig 5. Comparison of simulation results of risk indicators corresponding to different train headway in transfer to Platform 2 (side platform) in the up direction**

Fig 5 shows that the risk indicators, including total train operation delay and average passenger crowding degree on Platform 2, increase as the duration of the incident increases. However, there is little difference among the different impacts of headway *t* for both indicators. Therefore, it can be concluded that the train headway does not have a significant impact on the platform risk indicators. This is because the increase of train headway *t* will reduce the total transportation capability. However, owing to the large number of stranded passengers coming to Platform 2 at the early stage of an incident, the degree of platform crowding is faster in the former phase and slower in the latter phase. In other words, the [augment](file:///C:\Users\chenwy\AppData\Local\youdao\dict\Application\7.2.0.0703\resultui\dict\?keyword=augmented)ing extents of headway *t* increase are mild, which are paralleled with the results shown in Fig 6 Aggregating passengers (i.e., the crowding degree) will grow as train headway *t* increases but at a small rate [41].

In addition, Fig 5(b) demonstrates that the curves of the average platform passenger crowding degree are jaggedas the number of passengers suddenly increase and decrease as a result of loading and unloading when the train stops at the platform. Zhao et al. [42] observed that in a train operation cycle, the platform aggregated passenger variance rule is : increase sharply-to-decrease sharply and then decrease slowly-to-increase slowly. This result is roughly consistent with the cyclic trend of the simulation curve shownin Fig 5 (b).

**Fig 6. The effect of train headway on the number of** [**assembling passenger**](https://kns-cnki-net.webvpn.cueb.edu.cn/kcms/detail/knetsearch.aspx?dbcode=CJFD&sfield=kw&skey=urban%20traffic;assembling%20passenger%20number;Anylogic%20simulation;one-platform-transfer;arrival%20interval&code=&uid=WEEvREcwSlJHSldRa1Fhb09jT0pjWkNMcmk0dkdyd2RIWVJpRHRGYUptZz0=$9A4hF_YAuvQ5obgVAqNKPCYcEjKensW4IQMovwHtwkF4VYPoHbKxJw!!)**s at a platform**

**Discussion**

**Variance of risk indicators with passenger-arrival rate**

Fig 7 shows the risk indicators variance of train operation delay and platform crowding degree with passenger arrival rate φ. While Fig 7(a) shows the total up-direction train operation delay variance with incidental duration, Fig 7(b) shows Platform 2 average crowding degree variance. Herein, the passenger arrival rate φ is considered for passengers entering the platform through the inlet only. Those entering through transfer tunnels were excluded.

Fig 7(a) shows that train operation delay increases with incidental duration. In addition, the number of passengers coming from platform inlets increases (φ value is higher) at a higher rate. On the other hand, Fig 7(b) shows that before 900s the number of passengers and degree of crowding increase quickly，because the retention passenger swarm into Platform 2 sharply and train capacity is less. During the period of 900-1,100s, the crowding degree seems to slow down. After 1100s, all the retention passengers have arrived at Platform 2 and the passenger arrival rate decreases. However, so many significant interaction of passenger flows is observed, thereby reducing loading and unloading efficiency, further slowing down the speed of passengers departing the platform. Afterward, passenger crowding degree increases slowly.

**(a) Total up-direction train operation delay variance as a function of incidental duration**

**(b) Platform 2 average crowding degree variance as a function of incidental duration**

**Fig 7. Risk indicators variance of train operation delay and platform crowding degree with passenger-arrival rate *φ* in transfer to Platform 2 (up direction)**

Specifically, the crowding degree of up-direction Platform 2 tends to increase when φ≥3500, primarily due to the fact that the passenger arrival rate is more than the train maximum capacity. Consequently, the retention of passengers on Platform 2 increases. On the other hand, this value decreases and the speed of decline becomes faster when φ﹤3500 and T≥1800s. This can be attributed to the fact that the passenger arrival rate is less than train maximum capacity and passengers in Platform 2 are inclined to drop off. Hence, the single train operation delay of Line 2 will decrease, and the total passenger transportation capacity rises. As a result, the decreasing speed of the platform crowding degree accelerates.

**Comparison of risk indicators of propagation among metro networks**

As far as various structures of metro networks are concerned, their risk propagating process and rules are different as a result of network connecting rate discrepancy. To compare the incidental risk propagating rules of various networks, three typical networks are introduced as shown in Fig 8: a) grid, b) radial, and c) annular-radial structure. All networks have 4 lines, and their operation parameters are illustrated in Table 2.

**(a) Grid-type structure (b) Radial structure (c) Annular-radial structure**

**Fig 8. Different structure of metro network**

**Table 2. Operation parameters of lines on typical metro structures**

| Parameter | Train quantity of single-direction line | | | | Single train buffer time [s] | Transfer station failure buffer time [s] |
| --- | --- | --- | --- | --- | --- | --- |
|  | 1 | 2 | 3 | 4 |  |  |
| Value | 30 | 40 | 48 | 54 | 30 | 2400 |

Fig 9 displays the metro operation risk indicator change trends with incidental duration for the three structural networks. Fig 9(a) shows the affected line quantity with respect to the incidental duration. Fig 9(b) shows the suspension train quantity. For the sake of finding rules, an imaginary line was used to connect the discrete values of the results. In Fig 9, “*w*” represents the grid-type network, “*f*” represents the radial network, and “*h*” refers to the annular-radial network. Besides, the numbers “1-4” indicate Lines 1 to 4, respectively.

**(a) Affected lines quantity trends with incidental duration for the three structural networks**

**(b) Suspension train quantity trends with incidental duration for the three structural networks**

**Fig 9. Metro operation risk indicator change trends with incidental duration for the three structural networks**

For a grid-type network, as shown in Fig 9(a), we can see that two risk propagations through transfer stations are required for all lines to be suspended when the four lines are considered as initiating incidental lines, one at a time, and w2, w3, and w4 curves are identical as lines 2, 3, and 4 being the incidental line in order. For radial networks, only one risk propagation through transfer stations is needed for all lines suspension, also f2,f3, and f4 curves are identicalas line 2, 3, and 4 are being the initial lines orderly. Moreover, the affected line quantity trends of 4 lines as initiating incidental line for the annular-radial network are totally consistent with those for the radial network; therefore, f1 and h1 are represented by only one line and f2 and h2 are indicated by one line, and so on.

As can be seen from Fig 9(b), the suspension train quantity trends of the radial network as line 1-4 being the incidental lines one at a time are the same as those of the annular-radial network. Specifically, f1 and h1, f2 and h2, f3 and h3, and f4 and h4 coincide, respectively. Therefore, we used one line to represent the two networks as described above. All values on the curves increase rapidly during the period of 3600-4800s. The curves of the radial and annular-radial networks increase faster than those of the grid-type network. Moreover, after 4800s, the curve of the grid-type network increases slowly until the final state, when all lines are suspended, is reached.

It can be inferred that the risk indicators (including the affected line quantity and suspension train quantity) propagation speed of the grid-type network is relatively slow. In a grid, there are parallel lines whose connecting degree is relatively slow, and the incidental risk effect needs more time to propagate to the transfer station when all lines are suspended. Furthermore, grid-based networks resist destruction better than the other structures, but their passenger service efficiency is lower as more transfers are needed to arrive at a destination. On the hand, the risk propagation speed of the radial and annular-radial networks is relatively faster, because two arbitrary lines, in these networks, are mutually connected so that the incidental risk could spread to the whole network through only one transfer station propagation. The d[amage resistance ability](https://kns-cnki-net.webvpn.cueb.edu.cn/kcms/detail/knetsearch.aspx?dbcode=CJFD&sfield=kw&skey=destroy-resistant%20ability&code=&uid=WEEvREcwSlJHSldRa1Fhb09jT0pjWkNMcmNraDFreVRhNDI5K3J5R2xSQT0=$9A4hF_YAuvQ5obgVAqNKPCYcEjKensW4IQMovwHtwkF4VYPoHbKxJw!!) of these two networks is relatively worse. However, the passenger service efficiency is higher because fewer transfers are needed to reach a destination.

Concerning the condition that there is more than one transfer station between two lines, just one transfer station propagation is considered, thus causing the risk indicators curves of the radial and annular-radial networks to be consistent. In practice, as for annular-radial network, there is more than one transfer station between the annular lines and other lines, thus leading to faster risk propagation through all of the network. In summary, the order of three networks, from high to low, in terms of the incidental risk propagation speed is as follows: annular-radial, radial, and grid-type.

**Conclusion and limitations**

Rules of operation incidental risk propagation in a metro network under fully automatic operation mode were explored. The indicator computation models were developed and indicator variance rules for transfer station and different structural networks were discussed and verified through a simulation study. The computational models were applied and the network risk propagation rules were obtained. A summary of the conclusions from this study are presented as follows:

1. During the incidental risk propagation along a metro transfer station, there is a great effect on non-incidental line platform operation as detained passengers suddenly influx into the platform. Specifically, a train operation delay occurred at 450s for non-incident lines. Moreover, when passengers coming through the platform inlets are more (passenger arrival rate φ value is higher), the faster reaching the non-incidental line platform total train operation delay and the higher the crowding degree. However, train headway discrepancy has little influence on non-incidental line platform risk development.
2. Regarding the incidental risk propagation through a metro network, the propagation speed varies for differently structured networks. More specifically, the propagation speed of annular-radial network is the fastest, with the radial network being slower and the grid network being the slowest. Generally, the longer the incident line in network is (i.e., more operation trains on the line), the longer the duration of risk propagation along the network. Conversely, the shorter the line incidental line in network is (i.e., fewer operation trains on the line), the shorter the duration of the risk propagation along the network.

The conclusions as above are supposed to be supports for metro operation safety planning and network design. Although the profiles of incidental risk propagation along the metro network under fully automatic operations mode have been discussed in this paper, more studies need to be conducted to determine the effects of emergency dispatching measures on incidental operation risk propagation.

**References**

1. 2017 Annual Statistics and Analysis Report of Urban Rail Transit, Urban Rail Transit Association of China; 2018. Beijing, China.
2. Wan X., Li Q., Yuan J., Paul M. S.. Metro passenger behaviors and their relations to metro incident involvement. Accident Analysis and Prevention. 2015;82: 90-100.
3. Deng Y.. Research on assessment and control of urban metro network physical vulnerability [dissertation]. China: Southeast University; 2016.
4. Xu T., Liang Q., Chu B.. The security-risk management of Beijing subway network operation. Proceeding of the International Conference on Transportation Information and Safety; 2011; China. Wuhan;2011.
5. Ahmad F., Khan S. A.A.. Specification and verification of safety properties along a crossing region in a railway network control. Applied Mathematical Modelling. 2013;37: 5162-5170.
6. Kyriakidis M., Hirsch R., Majumdar A.. Metro railway safety: an analysis of accident precursors. Safety Science. 2012;50: 1535-1548.
7. Louie J., Shalaby A., Habib K. N.. Modelling the impact of causal and non-causal factors on disruption duration for Toronto's subway system: An exploratory investigation using hazard modelling. Accident Analysis and Prevention. 2017;98: 232-240.
8. Zhang X., Deng Y., Li Q., Skitmore M., Zhou Z. . An incident database for improving metro safety: The case of shanghai. Safety Science. 2016; 84:88-96.
9. Lu Y., Li Q., Xiao W. . Case-based reasoning for automated safety risk analysis on subway operation: case representation and retrieval. Safety Science. 2013;57: 75–-81.
10. Chen W., Zhang Y., Mohammad T. K., Geng Z.. Risk analysis on Beijing metro operation initiated from human factors. Journal of Transportation Safety & Security. 2019;11(6): 683-699.
11. Mine H., Kawai H.. Mathematics for Reliability Analysis. Asakurashoten;1982.
12. Iida Y., Wakabayashi H.. An approximation method of terminal reliability of road network using partial minimal path and cut sets. Proceeding of World Conference on Transport Policy;1989.
13. Sanso B., Soumis F.. Communication and transportation network reliability using routing models. IEEE Transactions on Reliability. 1991;40(1):29-38.
14. Bell M GH, Iida Y.. Transportation Network Analysis. John Wiley & Sons:New York;1997.
15. Latora V., Marchiori M.. Is the Boston subway a small-world network? Physica A: Statistical Mechanics & Its Applications. 2002;314(1):109-113.
16. Crucitti P., Latora V., Marchiori M., Rapisarda A.. Efficiency of scale-free networks: error and attack tolerance. Physica A: Statistical Mechanics & Its Applications. 2002; 320(C):622-642.
17. Han J., Guo J., Zhang X.. Reliability analysis of shanghai rail transit network. China Safety Science Journal. 2012;22(12): 103-108.
18. Luo Q., Yang Y., Mo Y., Li W., Zhang X.. Research on structural vulnerability of Shenzhen metro network based on complex network theory. In: Singapore 2018: Proceeding of the 3rd IEEE International Conference on Intelligent Transportation Engineering;p.18-22.
19. Angeloudis P., Fisk D.. Large subway systems as complex networks. Physica A: Statistical Mechanics and its Applications. 2006;367: 553-558.
20. Wang Z., Li Q., Liang Z.. Evaluation of urban metro network topological structure vulnerability. China Safety Science Journal. 2013;23(8): 114-119.
21. Yang Y., Liu Y., Zhou M., Li F., Sun C.. Robustness assessment of urban rail transit based on complex network theory: A case study of the Beijing Subway. Safety Science. 2015; 79: 149-162.
22. Diao P.. Analysis and simulation research on invulnerability of urban rail transit network[dissertation]. China: Beijing Jiaotong University; 2014.
23. Wu Z., Sun J., Xu R.. Calculating vulnerability index of urban metro systems based on satisfied route. Physica A: Statistical Mechanics and its Applications. 2019; 531:121722.
24. Jenelius E., Petersen T., Mattsson L. G.. Importance and exposure in road network vulnerability analysis. Transportation Research Part A Policy & Practice. 2006;40(7): 537-560.
25. Qu Y., Xu Z., Gong H., Huang Z., Wang P.. Vulnerability analysis of urban rail transit networks.Journal of Railway Science and Engineering. 2016;13(11):2276-2283.
26. Xu J., Song S., Zhai H., Chen M.. Study on construction of accident spread model for subway network based on vulnerability. Journal of Safety Science and Technology. 2017;13(3):96-101.
27. Assis W. O., Milani, Basílio E.A.. Generation of optimal schedules for metro lines using model predictive control. IFAC Proceedings Volumes. 2002; 35(1):475-480.
28. Castelli L., Pesenti R., Ukovich W.. Scheduling multimodal transportation systems. European Journal of Operational Research. 2004;155(3): 603-615.
29. Garcia R., Martin A.. Network equilibrium with combined modes: models and solution algorithms. Transportation Research Part B: Methodological. 2005;39(3): 223-254.
30. Silva R., Kang S. M., Airoldi E. M.. Predicting traffic volumes and estimating the effects of shocks in massive transportation systems. Proceedings of the National Academy of Sciences of the United States of America. 2015;112(18):5643-5648.
31. Chen L., Liu L., Niu L.. Research on the network congestion for large passenger flow of urban rail transit.Journal of Shijiazhuang Tiedao University (Natural Science Edition). 2014;2: 83-86.
32. Gao Y., Kroon L., Schmidt M., Yang L.. Rescheduling a metro line in an over-crowded situation after disruptions. Transportation Research Part B: Methodological. 2016;93: 425-449.
33. Jiao X.. The congestion propagation rules and coordinated inflow control method for urban rail transit network[dissertation]. China: Beijing Jiaotong University; 2016.
34. Zheng X.. Research on system dynamics modeling and congestion propagation control of passenger flow in urban rail transit network[dissertation]. China: Beijing Jiaotong University; 2017.
35. Huang W., Li H., Wang Y.. Passenger congestion propagation and control in peak hours for urban rail transit line. Journal of Railway Science and Engineering. 2017;14(1): 173-179.
36. Xiao W., Zhang Q... Modeling and simulation of congestion propagation based on disaster spreading dynamic model.Journal of Railway Science and Engineering.2018;15(6):1593-1600.
37. Chen W., Yang J., He S.. Research on risk propagation laws of subway operation sudden accident. Journal of Safety Science and Technology. 2018;14(7):86-91.
38. Code for design of metro (GB 50157-2013), Ministry of Housing and Urban-Rural Development of China.
39. Xu W., Wu Z.. Mathematical model for the passenger flow in subway station waiting rooms. Journal of Railway Science and Engineering. 2005;2(2):70-75.
40. Li Q., Li Q., Li Z.. An emergency evacuation routing optimization method based on space-time congestion concept. Acta Geodaetica et Cartographica Sinica. 2011; 40(4): 517-523.
41. Zhao M.. Research on the relationship between the urban rail transit platform and the departure intervals [dissertation]. China: Beijing Jiaotong University; 2011.
42. Zhao Y., Mao B., Yang Y., He T.. Methods of calculating the maximum assembling on urban rail transit platforms. Journal of Transportation Systems Engineering and Information Technology. 2011;11(2):149-154.

**Supporting information**

**S5(a) Table.** Fig 5. Comparison of simulation results of risk indicators corresponding to different train headway in transfer to Platform 2 (side platform) in the up direction

(a) Variance of total up-direction train operation delay of different headway *t* as a function of incidental duration

**S5(b) Table.** (b) Platform 2 average crowding degree variance of different headway *t* as a function of incidental duration

**S6 Table.** Fig 6. The effect of train headway on the number of [assembling passenger](https://kns-cnki-net.webvpn.cueb.edu.cn/kcms/detail/knetsearch.aspx?dbcode=CJFD&sfield=kw&skey=urban%20traffic;assembling%20passenger%20number;Anylogic%20simulation;one-platform-transfer;arrival%20interval&code=&uid=WEEvREcwSlJHSldRa1Fhb09jT0pjWkNMcmk0dkdyd2RIWVJpRHRGYUptZz0=$9A4hF_YAuvQ5obgVAqNKPCYcEjKensW4IQMovwHtwkF4VYPoHbKxJw!!)s at a platform

**S7(a) Table.** Fig 7. Risk indicators variance of train operation delay and platform crowding degree with passenger-arrival rate *φ* in transfer to Platform 2 (up direction)

(a)Total up-direction train operation delay variance as a function of incidental duration

**S7(b) Table.** (b)Platform 2 average crowding degree variance as a function of incidental duration

**S9(a) Table.** Fig 9. Metro operation risk indicator change trends with incidental duration for the three structural networks

(a)Affected lines quantity trends with incidental duration for the three structural networks

**S9(b) Table.** (b) Suspension train quantity trends with incidental duration for the three structural networks
